# 3-Hydroxybutyrate ameliorates insulin resistance by inhibiting PPARγ Ser273 phosphorylation in type 2 diabetic mice

**DOI:** 10.1038/s41392-023-01415-6

**Published:** 2023-05-26

**Authors:** Yudian Zhang, Zihua Li, Xinyi Liu, Xinyu Chen, Shujie Zhang, Yuemeng Chen, Jiangnan Chen, Jin Chen, Fuqing Wu, Guo-Qiang Chen

**Affiliations:** 1 School of Life Sciences, Tsinghua University, Beijing, 100084 P. R. China; 2 Department of Medical Genetics and Cell Biology, School of Basic Medical Science of Ningxia Medical University, Yinchuan, Ningxia 750004 P. R. China; 3 Center for Synthetic and Systems Biology, Tsinghua University, Beijing, 100084 China; 4 MOE Key Lab of Industrial Biocatalysis, Dept of Chemical Engineering, Tsinghua University, Beijing, 100084 P. R. China

**Keywords:** Biochemistry, Cell biology

## Abstract

3-Hydroxybutyrate (3HB) is a small ketone body molecule produced endogenously by the body in the liver. Previous studies have shown that 3HB can reduce blood glucose level in type 2 diabetic (T2D) patients. However, there is no systematic study and clear mechanism to evaluate and explain the hypoglycemic effect of 3HB. Here we demonstrate that 3HB reduces fasting blood glucose level, improves glucose tolerance, and ameliorates insulin resistance in type 2 diabetic mice through hydroxycarboxylic acid receptor 2 (HCAR2). Mechanistically, 3HB increases intracellular calcium ion (Ca^2+^) levels by activating HCAR2, thereby stimulating adenylate cyclase (AC) to increase cyclic adenosine monophosphate (cAMP) concentration, and then activating protein kinase A (PKA). Activated PKA inhibits Raf1 proto-oncogene serine/threonine-protein kinase (Raf1) activity, resulting in a decrease in extracellular signal-regulated kinases 1/2 (ERK1/2) activity and ultimately inhibiting peroxisome proliferator-activated receptor γ (PPARγ) Ser273 phosphorylation in adipocytes. Inhibition of PPARγ Ser273 phosphorylation by 3HB altered the expression of PPARγ regulated genes and reduced insulin resistance. Collectively, 3HB ameliorates insulin resistance in type 2 diabetic mice through a pathway of HCAR2/Ca^2+^/cAMP/PKA/Raf1/ERK1/2/PPARγ.

## Introduction

Diabetes mellitus is a chronic metabolic disease caused by absolute or relative deficiency of endogenous insulin or the body’s inability to efficiently utilize insulin. As the most common type of diabetes, T2D accounts for >90% of the total patients.^1^ Since it is mainly caused by insulin resistance (IR), the treatment of T2D can be carried out through reducing the level of IR or enhancing insulin sensitivity. First-line drugs for T2D, such as metformin and insulin sensitizer thiazolidinediones (TZDs), can reduce insulin resistance in liver, skeletal muscle and adipose tissues.^2,3^ However, both drugs have their limitations and side effects. More specifically, metformin can cause diarrhea, nausea and other intestinal adverse reactions, as well as deficiency of vitamin B12;^3^ TZDs may cause fluid retention (edema, heart failure), body weight increases, bladder cancer (mainly caused when using pioglitazone) and congestive heart failure.^4^ Therefore, it is urgent to develop safer and more effective drugs for the treatment of T2D, which has practical significance for global patients and international public health.

Peroxisome proliferator-activated receptor γ (PPARγ) is the target of TZDs, but the occurrence of side effects is also attributed to the over activation of PPARγ transcriptional activity via TZDs.^5^ The post translational modification of PPARγ regulating its activity, is not only a new method to optimize the function and reduce side effects of PPARγ, but also a research hotspot for new drug development.^6^ For instance, a non-agonist ligand for PPARγ, SR1664, could block cyclin-dependent kinase 5 (CDK5) and MEK/ERK mediated PPARγ phosphorylation on its Ser273 site, and enhance insulin sensitivity in mice without common side effects like fluid retention and body weight increases.^7,8^

3HB is the main component of ketone bodies, which could become an energy resource when there is a lack of blood glucose. Except for its function as a passive energy carrier, an increasing number of studies have proven that 3HB could participate in cell signal transduction as a signal molecule.^9^ As an endogenous ligand of hydroxycarboxylic acid receptor 2 (HCAR, also known as G protein coupled receptor 109a, GPR109a), 3HB plays an important role in tissues expressing HCAR2.^10^ HCAR2 is a type of G_i/o_ protein coupled receptor, mediating G_i/o_ protein to inhibit the activity of adenylate cyclase (AC) and reduce the level of cyclic adenosine monophosphate (cAMP), thereby reducing the activity of cAMP-dependent kinase A (PKA).^11^ On the contrary, in certain cells, HCAR2 agonist 3HB and nicotinic acid can increase the intracellular Ca^2+^ concentration by G_βγ_ subunit to activate phospholipase C_β_.^12^ The elevated intracellular Ca^2+^ concentration could activate AC, resulting in increased cAMP concentration and activation of cAMP/PKA pathway.^13,14^

Human experiments have shown that 3HB could reduce the blood glucose level of T2D patients.^15^ However, there are not yet systematic studies to evaluate the therapeutic effect of 3HB on T2D and not yet clear mechanisms to explain the hypoglycemic function. This study filled the above gaps by systematically elaborating the therapeutic effect of 3HB, and confirmed that 3HB is a safe and effective agent that can improve insulin resistance. At the same time, this paper further explored the molecular mechanism of 3HB decreasing insulin resistance level, which is by indirectly inhibiting the phosphorylation of PPARγ Ser273 site through HCAR2/Ca^2+^/cAMP/PKA/Raf1/ERK1/2/PPARγ signaling pathway.

## Results

### 3HB treatment reduced insulin resistance in T2D mice

To explore the effect of 3HB on T2D, leptin receptor deficiency mice (db/db) and streptozotocin (STZ) induced T2D mice were used.^16^ 1,3-butanediol (1,3-BDO), a precursor of endogenous 3HB, could be efficiently converted to 3HB in the liver.^17,18^ Considering the effect of increasing blood 3HB concentration and the influence on fluid intake volume of mice, 10% 1,3-BDO aqueous solution was chosen as drinking fluid for the mice to maintain a blood 3HB level of over 1.5 mM based on the literatures and corresponding experimental results (Supplementary Fig. S5b).^19,20^

**Fig. 1:**
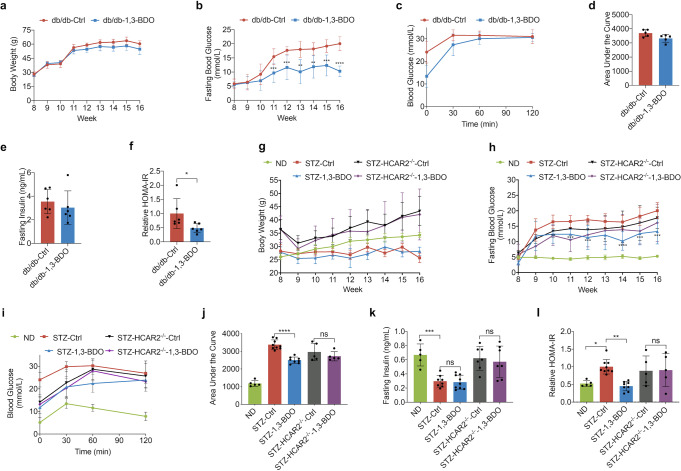
3HB Treatment Reduces Insulin Resistance in T2D Mice. **a** Body weights of db/db mice (*n* = 10). **b** Overnight fasted blood glucose level of db/db mice (*n* = 10). **c** The glucose tolerance test of db/db mice. Glucose (2 g/kg body weight) was intraperitoneally (i.p.) administered in overnight-fasted db/db mice. (*n* = 5) **d** The area under the curve (AUC) in **c**. **e** Fasting serum insulin levels of db/db mice after 8 weeks of indicated treatment. (*n* = 6) **f** A homeostasis model for assessment of the insulin resistance index (HOMA-IR) of the mice in **e**. **g** Body weights of STZ induced T2D mice. (*n* = 10) **h** Overnight fasted blood glucose level of STZ mice. (*n* = 10) **i** The glucose tolerance test of STZ mice. Glucose (2 g/kg body weight) was intraperitoneally (i.p.) administered in overnight-fasted STZ mice. (*n* = 5–9) **j** The area under the curve (AUC) in **i**. **k** Fasting serum insulin levels of STZ mice after 8 weeks of indicated treatment. (*n* = 5–9). **l** A homeostasis model for assessment of the insulin resistance index (HOMA-IR) of STZ mice. (*n* = 5–9) Data reported as mean ± SD, *p < 0.05, **p < 0.001, and ***p < 0.0001

Vehicle-treated db/db mice (db/db-Ctrl) developed pronounced hyperglycemia, with fasting blood glucose (FBG) levels ~18 mM (Fig. 1b). 10% 1,3-BDO treated mice (db/db-1,3-BDO) showed a significant reduction in FBG, which was <12 mM (Fig. 1b). The improvement in glucose homeostasis was achieved without a significant change in the body weight (Fig. 1a). Intraperitoneal glucose tolerance test (IPGTT) was performed to evaluate the body’s functionality of controlling blood glucose. While db/db-1,3-BDO mice did not show improved glucose clearance compared to db/db-Ctrl mice (Fig. 1c, d). Meanwhile, 3HB treatment did not change the fasting serum insulin level (Fig. 1e). However, the calculated results of HOMA-IR, a homeostatic model of insulin resistance, showed that 3HB could significantly reduce HOMA-IR by 52% (Fig. 1f), indicating that 3HB effectively reduced insulin resistance levels in db/db mice.

C57BL/6 J and HCAR2^-/-^ (C57BL/6 J background) male mice treated with high-fat diet combined with low doses of STZ progressively lost glucose control compared to normal diet mice (ND) (Fig. 1h). The FBG in the ND group was always maintained within the normal range of 3.9–6.1 mM, while the FBG of the STZ-Ctrl group gradually increased and tended to be stabilized above 16 mM. Compared with STZ-Ctrl, the FBG of STZ-1,3-BDO mice was consistently and significantly lower than that of STZ-Ctrl group, and the highest value was just 13.3 mM (Fig. 1h). STZ-HCAR2^-/-^ mice consistently had lower FBG than wild-type T2D mice (Fig. 1h), which was attributed to the effect of HCAR2 deficiency. However, limited to no benefit on FBG was provided by 1,3-BDO treatment in STZ-HCAR2^-/-^ mice. The mean FBG of STZ-HCAR2^-/-^-1,3-BDO group seemed lower than that of STZ-HCAR2^-/-^-Ctrl, but without statistical difference. Therefore, even though the knockout of HCAR2 resulted in the lower FBG in STZ-HCAR2^-/-^ mice, it could still be found that HCAR2 may mediate the hypoglycemic effect of 3HB on FBG in STZ-induced T2D mice to some degree. When comparing the weight changes of mice in each group, the body weight of ND group increased constantly over time. Although fed on a high-fat diet, both STZ-Ctrl and STZ-1,3-BDO mice body weights did not change significantly during the studies (Fig. 1g). The body weights of HCAR2^-/-^ mice were significantly higher than that of wild-type mice, which was consistent with the reported phenomenon of HCAR2^-/-^ mice.^21^ Unlike in db/db mice, 1,3-BDO treatment showed improved glucose clearance compared to STZ-Ctrl mice in IPGTT (Fig. 1i, j). After the glucose injection, the blood glucose levels of mice in the STZ-1,3-BDO group were significantly lower than those in the STZ-Ctrl group. The blood glucose levels of both STZ-HCAR2^-/-^ groups were consistently lower than those in STZ-Ctrl group, but the effect of 1,3-BDO was not reflected (Fig. 1i, j). Compared with the ND group, the fasting insulin of the STZ-Ctrl and STZ-1,3-BDO groups was significantly reduced by >55%, but there was no difference between the two groups, indicating that 1,3-BDO had no effect on the level of serum insulin (Fig. 1k). Surprisingly, the serum fasting insulin levels of the two STZ-HCAR2^-/-^ groups were almost the same as that of ND group, and also with no inter-group difference (Fig. 1k). This suggested that HCAR2 deletion may cause an impact on insulin expression or secretion in mice, and also explained the reason why STZ-HCAR2^-/-^ mice had lower blood glucose levels than wild-type STZ-induced T2D mice did (Fig. 1h). The calculated results of HOMA-IR showed that the level of insulin resistance in the STZ-Ctrl group was increased by about 48% compared to that of the ND group, while 3HB provided 55% reduction of HOMA-IR value in STZ-1,3-BDO group compared to STZ-Ctrl mice (Fig. 1l). Although STZ-HCAR2^-/-^ mice had lower FBG levels (Fig. 1h), it was attributed to the effect of higher levels of insulin in STZ-HCAR2^-/-^ mice. Therefore, the insulin resistance levels in STZ-HCAR2^-/-^ mice were similar to that of STZ-Ctrl group, and 1,3-BDO had no significant effect on insulin resistance in STZ-HCAR2^-/-^ mice (Fig. 1l).

Apart from the findings above, 1,3-BDO treatment reduced adipose tissue, liver and kidney damage caused by T2D, and improved blood lipids and liver functions (Supplementary Fig. S2-S4). The protective effect on adipose tissue and liver was mediated by HCAR2 (Supplementary Fig. S3). In combination, in vivo application of 1,3-BDO resulted in reduced FBG, insulin resistance level and tissue injury through HCAR2 in T2D mice, strongly supporting the potential therapeutic value of 3HB in T2D.

### 3HB regulated Ca^2+^, cAMP, ERK1/2 and PPARγ related biological processes or signaling pathways

**Fig. 2:**
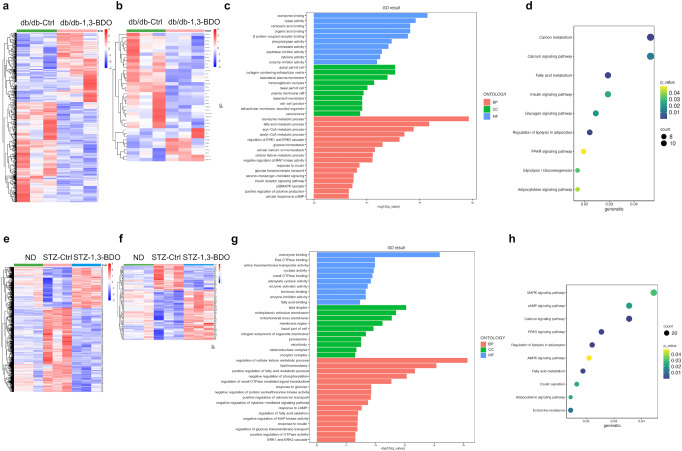
Transcriptomics Analysis for Adipose Tissue of T2D Mice. **a** After 8 weeks of indicated treatment, the adipose tissue of db/db mice was collected and studied the differential gene expression analysis (*p* < 0.05) by RNA sequencing. **b** Downstream genes of PPARγ in differentially expressed genes of db/db mice. **c** Gene ontology (GO) enrichment analysis of db/db mice. The related biological processes (BP), cellular component (CC) and molecular function (MF) were analyzed. **d** Kyoto Encyclopedia of Genes and Genomes (KEGG) enrichment analysis of db/db mice. **e** After 8 weeks of indicated treatment, the adipose tissue of STZ induced T2D mice was collected and conducted the differential gene expression analysis (*p* < 0.05) by RNA sequencing. **f** Downstream genes of PPARγ in differentially expressed genes of STZ mice. **g** GO enrichment analysis of STZ mice. The related biological processes (BP), cellular component (CC) and molecular function (MF) were analyzed. **h** KEGG enrichment analysis of STZ mice

To further determine the underlying molecular mechanism of 1,3-BDO treatment in T2D mice, we performed RNA-sequencing to detect the gene expression profiles of adipose tissue of each group, and cluster maps were drawn according to differentially expressed genes (Fig. 2a, e). There were 800 differentially expressed genes in adipose tissue samples of mice in db/db-Ctrl group and db/db-1,3-BDO group. The heatmap (red and blue colors indicate increased and decreased gene expressions, respectively) showed that 1,3-BDO treatment could significantly alter the gene expression pattern of adipose tissue in db/db mice (Fig. 2a). 2461 differentially expressed genes were detected in the adipose tissue of ND, STZ-Ctrl, and STZ-1,3-BDO groups. Compared with STZ-Ctrl group, 1,3-BDO could alter the gene expression pattern of adipose tissue in T2D mice and promote the reversal of it to that of ND mice (Fig. 2e).

Related enriched Gene Ontology (GO) terms were shown in Fig. 2c, g. In terms of biological processes (BP), 3HB was involved in Ca^2+^ and cAMP related processes, glucose homeostasis, insulin signaling pathways and extracellular signal-regulated kinases 1/2 (ERK1/2) regulation of the MAPK signaling pathway in adipose tissue.

The KEGG enrichment results showed that the regulation of PPAR, MAPK, Ca^2+^, cAMP, adipokines, insulin and some other signaling pathways were affected by 1,3-BDO administration (Fig. 2d, h). PPARγ is the main type of PPAR in adipose tissue, and is a major target for drug development in T2D,^22,23^ suggesting that 3HB could act as an insulin resistance reduction agent through PPARγ signaling pathway. Meanwhile, cAMP and Ca^2+^ can be the two important second messengers in the possible cell signal transductions caused by 3HB.

PPARγ regulates the transcription of multiple genes involved in adipogenesis and glucose homeostasis.^24^ If 1,3-BDO treatment has a regulatory effect on PPARγ-related signaling pathways in adipose tissue, the expression of PPARγ regulated downstream genes should also be affected. We screened a total of 39 reported PPARγ downstream genes from all differentially expressed genes of db/db mice, and performed cluster analysis on the screening results. 1,3-BDO treatment significantly changed the expression of PPARγ downstream genes in adipose tissue of db/db mice (Fig. 2b). A total of 136 reported PPARγ regulated genes were found out from all differentially expressed genes of ND, STZ-Ctrl, and STZ-1,3-BDO groups, and the cluster analysis on the screening results showed that the occurrence of T2D caused great changes in the expression of PPARγ downstream genes. However, 1,3-BDO treatment changed the expression pattern of PPARγ downstream genes in adipose tissue of T2D mice, and made it closer to that of ND group (Fig. 2f). In HCAR2^-/-^ groups, the difference of total differentially expressed genes and PPARγ downstream genes patterns between STZ-HCAR2^-/-^-Ctrl and STZ-HCAR2^-/-^-1,3-BDO groups was not significant (Supplementary Fig. S1a and S1b). In summary, 1,3-BDO treatment affects the expression of PPARγ controlling downstream genes via HCAR2 in adipose tissues.

### 3HB inhibits phosphorylation of PPARγ Ser273 in vitro and in vivo

**Fig. 3:**
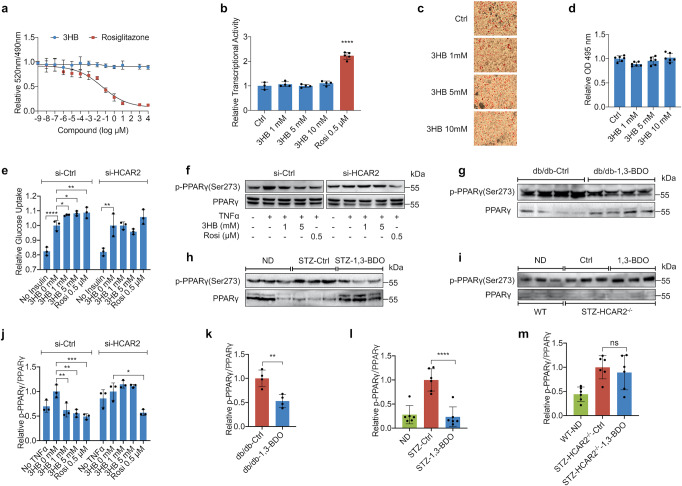
3HB Inhibits Phosphorylation of PPARγ Ser273 in vitro and in vivo. **a** TR-FRET PPARγ ligand displacement assay of 3HB and positive control rosiglitazone. **b** Influence on PPARγ transcriptional activity of 3HB or 0.5 μM rosiglitazone was measured using a 293 T cells based on the luciferase reporter assay. **c** Oil red-O staining of mature 3T3-L1 adipocytes after 8 days of 3HB treatment. **d** Quantification of the staining results in **c** was presented relative to the control. **e** 3T3-L1 adipocytes with or without siRNA knockdown of HCAR2 were pretreated with 3HB or 0.5 μM rosiglitazone for 24 h. After 10 min stimulation of insulin, the glucose uptake of cells was measured by a bioluminescent assay. **f** 3T3-L1 adipocytes with or without siRNA knockdown of HCAR2 were treated with TNFα, followed by treatment with 3HB or rosiglitazone (Rosi) for 1 h. Phosphorylated PPARγ at Ser273 and total PPARγ were detected using anti-pPPARγ or anti-PPARγ antibodies, respectively. **h**–**i** pPPARγ (Ser273) and total PPARγ of adipose tissue in 8-week treated db/db mice, STZ induced T2D mice and STZ induced HCAR2^-/-^ T2D mice, respectively. **j**–**m** The amounts of pPPARγ (Ser273) and total PPARγ in **f**–**i** were quantified using ImageLab, and pPPARγ/ PPARγ ratio was calculated. The results (expressed as the mean ± SD, *n* ≥ 3) were presented relative to the control (3HB 0 mM, db/db-Ctrl, STZ-Ctrl or STZ-HCAR2^-/-^-Ctrl), *p < 0.05, **p < 0.001, and ***p < 0.0001, **** *p* < 0.00001

1,3-BDO treatment affects the transcriptional level of PPARγ downstream target genes through HCAR2 (Fig. 2b and f, Supplementary Fig. S1a and S1b). PPARγ agonists are widely used in the treatment of T2D and related complications by enhancing insulin sensitivity.^2,25^ To investigate the exact effect of 3HB on PPARγ, the LanthaScreen TR-FRET PPARγ Competitive Binding Assay Kit was used to investigate whether 3HB is a PPARγ ligand. The IC_50_ of rosiglitazone (Rosi) in this assay was calculated to be 37 nM, which is consistent with reported literature,^26^ but 3HB could not competitively bind to PPARγ ligand binding domain, indicating that 3HB is not a ligand of PPARγ (Fig. 3a). Meanwhile, 3HB had no effect on PPARγ transcriptional activity (Fig. 3b). PPARγ regulates the transcription of genes involved in lipogenesis, and PPARγ agonists or inhibitors could promote or inhibit the differentiation of preadipocytes.^2,24^ However, 3HB could not affect the differentiation of 3T3-L1 preadipocytes (Fig. 3c, d). To sum up, 3HB has no direct action on PPARγ. Whereas, in 3T3-L1 adipocytes, 3HB could significantly promote insulin-dependent glucose uptake with a weak dose-dependent effect after 24 h treatment. While in the HCAR2 knockdown group, the promoting effect of 3HB disappeared (Fig. 3e), indicating that 3HB might enhance insulin sensitivity in adipocytes via HCAR2 mediation.

In fact, ligands of PPARγ, such as TZDs, may cause overactivation of PPARγ, leading to obesity, bone loss, heart failure and other serious side effects.^4^ Posttranslational modification of PPARγ is a new drug research direction to optimize the function of PPARγ and avoid adverse reactions.^27^ For example, PPARγ serine 273 site can be phosphorylated by CDK5 or MEK/ERK, resulting in functional disorder of PPARγ, inhibiting the expression of genes related to insulin sensitivity, and finally leading to insulin resistance.^8,28^ Inhibition of phosphorylation at this site significantly improves insulin resistance while avoiding the side effects associated with over-activation of PPARγ.^8,28^ To explore the effect of 3HB on the phosphorylation of PPARγ Ser273 and the role of HCAR2 in this process, 3T3-L1 adipocytes transfected with si-Ctrl or si-HCAR2 interfering RNA were stimulated with TNFα for 1 h to increase the cellular phosphorylation level of PPARγ Ser273, and followed with 3HB or Rosi treatment for another 1 h. The phosphorylation level of PPARγ Ser273 was detected by Western blot analysis, and the ratio of phosphorylated PPARγ (p-PPARγ)/total PPARγ was calculated to reflect the phosphorylation level of PPARγ Ser273. Both 3HB and Rosi could significantly inhibit the phosphorylation of PPARγ Ser273. However, when HCAR2 was silenced, the inhibitory effect of 3HB on PPARγ Ser273 phosphorylation disappeared (Fig. 3f). The results of quantitative analysis also showed that 1 mM and 5 mM 3HB effectively inhibited the phosphorylation level of PPARγ Ser273 by 38 and 44%, respectively, which was in a dose-dependent manner and even lower than the non-TNFα group (Fig. 3j). Therefore, 3HB could inhibit PPARγ Ser273 phosphorylation through HCAR2 in 3T3-L1 adipocytes.

In order to investigate whether 1,3-BDO treatment inhibits the phosphorylation of PPARγ Ser273 in the adipose tissue of T2D mice, the total protein of the adipose tissue of db/db mice was extracted for Western blot detection. 1,3-BDO treatment indeed reduced the phosphorylation of PPARγ Ser273 in the adipose tissue of db/db mice, along with significantly increased total amount of PPARγ (Fig. 3g). Quantitative analysis showed that 1,3-BDO treatment reduced the phosphorylation level of PPARγ Ser273 in adipose tissue by 47% (Fig. 3k). Compared with healthy mice in the ND group, the total protein expression of PPARγ in the adipose tissue of STZ-Ctrl was decreased, yet the phosphorylation of PPARγ Ser273 was significantly increased. 1,3-BDO treatment promoted the expression of PPARγ in the adipose tissue of STZ-1,3-BDO mice, together with a 76% reduction in PPARγ Ser273 phosphorylation (Fig. 3h and 3l). In HCAR2 knockout T2D mice, the inhibitory effect of 3HB on PPARγ Ser273 phosphorylation was abolished (Fig. 3i, m), suggesting that HCAR2 was essential for 3HB to inhibit PPARγ Ser273 phosphorylation in adipose tissue of T2D mice.

### 3HB inhibited phosphorylation of PPARγ Ser273 via HCAR2/Ca^2+^/cAMP/PKA/Raf1/ERK1/2/PPARγ pathway

**Fig. 4:**
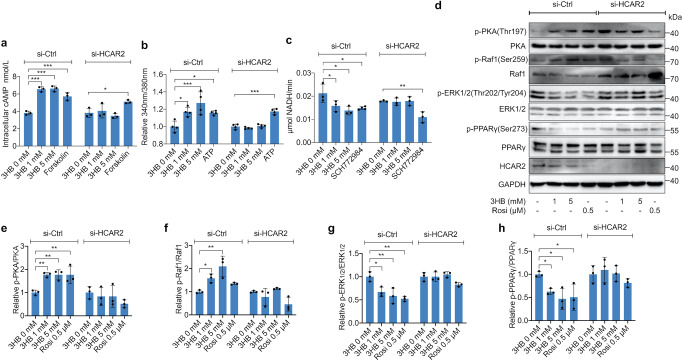
3HB Inhibits Phosphorylation of PPARγ Ser273 Through HCAR2/Ca^2+^/cAMP/PKA/Raf1/ERK1/2 Pathway. **a** 3T3-L1 adipocytes with or without siRNA knockdown of HCAR2 were treated with 3HB or 100 μM forskolin for 1 h. Intracellular cAMP concentration was detected using an ELISA kit. **b** Intracellular Ca^2+^ levels of 3T3-L1 adipocytes treated with 3HB or 1 μM ATP for 1 h were detected with Fura-2/AM probe kit. The results were presented relative to 3HB 0 mM. **c** The kinase activity of ERK1/2 of 3T3-L1 adipocytes treated with 3HB or 10 μM SCH779284 for 1 h was measured using an ERK1/2 kinase Activity Quantitative detection Kit. **d** 3T3-L1 adipocytes with or without siRNA knock down of HCAR2 were treated with 3HB or 0.5 μM rosiglitazone (Rosi) for 1 h. Phosphorylated PKA at Thr197, Raf1 at Ser259, ERK1/2 at Thr202/Tyr204, PPARγ at Ser273, and total PKA, Raf1, ERK1/2, PPARγ, HCAR2, GAPDH were studied using corresponding antibodies. **e**–**h** The amounts of phosphorylated and total proteins in **d** were quantified using ImageLab, and pPKA/PKA, pRaf1/Raf1. pERK1/2/ERK1/2 and pPPARγ/ PPARγ ratio was calculated. The results (expressed as the mean ± SD, *n* = 3) were presented relative to 3HB 0 mM, *p < 0.05, **p < 0.001, and ***p < 0.0001

Previous studies had shown that ERK1/2 was involved in the phosphorylation of PPARγ Ser273, and its inhibitors can significantly decrease PPARγ Ser273 phosphorylation level in adipose tissue of obese mice.^8^ The activation of HCAR2 can increase intracellular Ca^2+^ concentration in macrophages and hippocampal neurons, which then activates AC, and in turn increases intracellular cAMP levels.^13,29^ The above results showed that 3HB inhibited PPARγ Ser273 phosphorylation via HCAR2 and enhanced insulin sensitivity. To verify if cAMP, Ca^2+^ and ERK1/2 were involved in this process, 3T3-L1 adipocytes transfected with si-Ctrl or si-HCAR2 interfering RNA were treated with different concentrations of 3HB or 100 μM forskolin for 1 h, and then the intracellular cAMP concentration was studied. 3HB could significantly increase the cAMP level to >6.5 nmol/L. When HCAR2 expression was knocked down, the effect of 3HB was abolished (Fig. 4a), suggesting that 3HB could increase intracellular cAMP level in adipocytes through HCAR2. 3HB could also significantly increase the levels of intracellular Ca^2+^ through HCAR2 in a dose-dependent manner after 1 h treatment (1 mM treated group could increase by 16.9%; 5 mM group by 27.1%) (Fig. 4b). In order to explore whether 3HB affects the activity of ERK1/2 or not, we treated 3T3-L1 adipocytes with 3HB or 10 μM ERK1/2 inhibitor SCH772984 for 1 h, and then the ERK1/2 activity was detected, which was reflected by the final NADH production efficiency of the ERK1/2 catalytic reaction system. 3HB, like the ERK1/2 inhibitor SCH772984, could effectively inhibit ERK1/2 activity in a dose-dependent manner (1 mM 3HB could reduce ERK1/2 activity by~26%; 5 mM by~35%). When the expression of HCAR2 was knocked down by siRNA, the inhibitory effect of 3HB on ERK1/2 activity also disappeared (Fig. 4C), demonstrating that 3HB could inhibit ERK1/2 activity in adipocytes via HCAR2.

Studies have shown that protein kinase A (PKA) activated by cAMP can phosphorylate the Ser259 site of Raf1 proto-oncogene serine/threonine-protein kinase (Raf1), the upstream kinase of MEK and ERK1/2, and the phosphorylation of Ser259 will lead to the inactivation of Raf1. Inactive Raf1 cannot activate MEK, which eventually leads to the inhibition of ERK1/2 activity.^30–32^ 3HB could increase the concentration of cAMP in 3T3-L1 adipocytes and inhibit the activity of ERK1/2. Thus, these two effects of 3HB are likely to be linked by a putative pathway: cAMP/PKA/Raf1/ERK1/2. Total protein of 3T3-L1 adipocytes treated with 3HB or 0.5 μM Rosi for 1 h was extracted for Western blot assays of phosphorylation of PKA (Thr197), ERK1/2 (Thr202/Tyr204), Raf1 (Ser259) and PPARγ (Ser273). 3HB significantly increased the phosphorylation levels of PKA (Thr197) and Raf1 (Ser259), but decreased ERK1/2 (Thr202/Tyr204) and PPARγ (Ser273) phosphorylation in adipocytes. However, these effects were all abolished in HCAR2 silenced adipocytes (Fig. 4d–h). In other words, 3HB effectively activated PKA, inhibited Raf1, reduced the activity of ERK1/2 and the phosphorylation level of PPARγ Ser273 via HCAR2.

To sum up, 3HB could increase intracellular Ca^2+^ and cAMP levels via HCAR2 to activate PKA, thereby inhibiting Raf1 activity, resulting in a decrease in ERK1/2 activity, and ultimately decreasing the phosphorylation level of PPARγ Ser273. The pathway of 3HB/HCAR2/Ca^2+^/cAMP/PKA/Raf1/ERK1/2/PPARγ might be the molecular mechanism by which 3HB ameliorates insulin resistance.

### 3HB regulated the expression of genes affected by PPARγ Ser273 phosphorylation in 3T3-L1 adipocytes

**Fig. 5:**
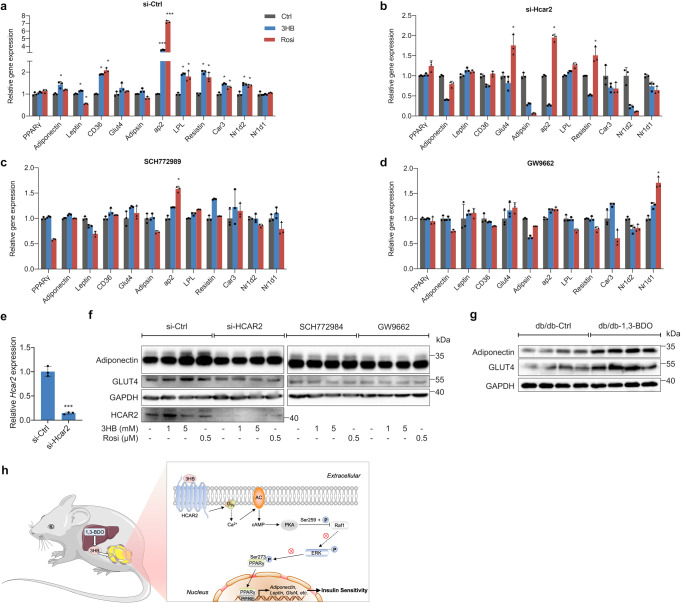
3HB Affects the Expression of Genes Regulated by PPARγ Ser273 Phosphorylation in 3T3-L1 Adipocytes. **a**, **b** Gene expression of adipocytes with or without siRNA knockdown of HCAR2 after 6 h 5 mM 3HB or 0.5 μM rosiglitazone (Rosi) treatment. **c**, **d** Gene expression of adipocytes treated with 5 mM 3HB or 0.5 μM for 6 h in the presence of 10 μM SCH779284 or 20 μM GW9662. **e** Relative *HCAR2* expression of adipocytes with or without siRNA knockdown of HCAR2. **f** 3T3-L1 adipocytes with or without siRNA knockdown of HCAR2 were treated with 3HB or 0.5 μM rosiglitazone (Rosi) for 48 h in the absence or presence of 10 μM SCH779284 or 20 μM GW9662. Adiponectin, GLUT4, HCAR2 and GAPDH were detected by corresponding antibodies. **g** Adiponectin and GLUT4 protein expression of db/db mice adipose tissue. **h** Schematic diagram of the mechanism by which 3HB ameliorates insulin resistance in type 2 diabetic mice. The results (expressed as the mean ± SD, *n* = 3) were presented relative to Ctrl groups for each gene, *p < 0.05, and ***p < 0.0001

The expression levels of PPARγ regulated downstream genes in adipocytes was studied using the real-time quantitative PCR, and several genes affected by phosphorylation of PPARγ Ser273 were also chosen.^28^ Although 3HB could not change the transcriptional level of PPARγ, it had a significant regulatory effect on the expression of PPARγ regulated genes, which was similar to Rosi (Fig. 5a). HCAR2 siRNA could effectively inhibit the transcription of *Hcar2* by ~86% (Fig. 5e), while 3HB had no effect on the transcription of PPARγ downstream genes in these adipocytes, the effect of Rosi still existed (Fig. 5b). When inhibiting ERK1/2 kinase activity or antagonizing PPARγ in adipocytes with SCH772984 or GW9662, the effects of 3HB and Rosi on the expression of most selected genes were abolished (Fig. 5c, d). The above results suggested that 3HB could regulate the transcription of PPARγ downstream genes affected by Ser273 phosphorylation via HCAR2 and ERK1/2 in 3T3-L1 adipocytes, further revealing that the effect of 3HB on the phosphorylation of PPARγ Ser273 was depending on HCAR2 and ERK1/2.

At the protein expression level, 3HB also promoted the expression of PPARγ regulated downstream genes encoding adiponectin and glucose transporter 4 (GLUT4), while the deficiency or inhibition of HCAR2, ERK1/2 or PPARγ eliminated the effect of 3HB (Fig. 5f). The promotion effect of 3HB on adiponectin and GLUT4 protein expression was also verified in adipose tissue of db/db T2D mice (Fig. 5g).

Therefore, 3HB significantly regulated the expression of PPARγ downstream genes which were affected by Ser273 phosphorylation at both gene transcription and protein translation levels, resulting in reducing of insulin resistance level. This effect was dependent on HCAR2 and ERK1/2, further confirming the above molecular mechanism by which 3HB inhibited PPARγ Ser273 phosphorylation.

## Discussion

With the increasing number of patients of T2D around the world, the problems of side effects and high prices of treatment drugs gradually emerged and became more and more acute.^1^ Discovering and developing new drugs with low or even no side effect is of great significance. 3HB is a small molecule derived from human ketone body with a relatively wide range of safe dosages.^33^ This study systematically elaborated the therapeutic effect of 3HB in T2D mice, and found a novel molecular mechanism by which 3HB ameliorates insulin resistance. Based on present in vitro results, we propose for the first time that 3HB regulates ERK1/2 activity via HCAR2/Ca^2+^/cAMP/PKA/Raf1, optimizing PPARγ function from the aspect of post-translational modification to reduce insulin resistance.

1,3-BDO was used as a safe food additive since 1960s, and studies revealed that it could be metabolized into 3HB in the liver to provide energy.^18^ Solution of 1,3-BDO was used for drinking to maintain a high blood concentration of 3HB.^19,20^ In this study, 10% 1,3-BDO solution was provided for healthy C57BL/6 J mice to drink freely for 2 h. The final blood concentration of 3HB was ~1.6 mM (Supplementary Fig. S5b), much lower than that of patients with ketoacidosis (9.1 mM).^34^ Therefore, exogenous supplement of 3HB by drinking 1,3-BDO had better effect and higher security for elevating blood ketone without further introduction of excessive salt ions compared to oral ministration of 3HB-Na (Supplementary Fig. S5a). The mean blood 3HB of db/db-Ctrl mice increased to 2.27 mM after fasting for 12 h, while db/db-1,3-BDO mice had a significantly higher blood 3HB level of 3.83 mM (Supplementary Fig. S5c). ND healthy mice, STZ-Ctrl and STZ-1,3-BDO groups had an average fasting blood 3HB levels of 1.2 mM, 1.43 mM and 2.3 mM, respectively (Supplementary Fig. S5d). The concentration of blood 3HB in HCAR2^-/-^T2D mice had the same increasing trend as wild-type mice (Supplementary Fig. S5d). The fasting blood 3HB of all groups did not exceed 4 mM, indicating that 1,3-BDO could be used as a safe 3HB supplement for T2D patients. Hyperglycemia could cause irreversibly damage to many organs.^1^ 3HB has excellent protective effects on cardiovascular system, central nervous system, kidney, retina and other tissues.^20,35–39^ This study found that 3HB could reduce the pathological damage of adipose tissue, liver and kidney caused by T2D (Supplementary Fig. S2-S3), and improve blood lipid and liver function in T2D mice (Supplementary Fig. S4). Therefore, 3HB in the treatment of T2D is beneficial to protect the functions of organs and tissues from T2D related complications. Lipotoxicity plays a key role in the development of both insulin resistance,^40^ the improvement of blood lipid in T2D mice may also be the mechanism by which 3HB reduces insulin resistance, which is worthy of further study.

Our study showed the ameliorative effect of 3HB on insulin resistance in T2D mice, but it would be better to have more in vivo evidence to demonstrate the effect of 3HB on insulin sensitivity, such as insulin tolerance test, euglycemic glucose clamp or detection of AKT phosphorylation in adipose tissue. It was reported that 3HB treatment increased AKT phosphorylation in mouse muscle.^41,42^ We speculated that 3HB could have a similar effect in T2D mice, and further studies are needed to verify the hypothesis. Limitations of this study also include the fact that the molecular mechanism was only accomplished at the cellular level and future in vivo experiments are required to verify the conclusions.

In this study, the effect of 3HB on the activity of CDK5 was also detected. CDK5 needs to combine with the regulatory factor p35 in cells to form a CDK5/p35 complex before playing the role of kinase. Afterwards, p35 was cleaved by calpain, forming a CDK5/p25 complex for stronger and longer activity.^43^ 3HB could significantly inhibit the activity of CDK5/p35 as Roscovitine, a CDK5/p35 inhibitor (Supplementary Fig. S6a), without making any impact on CDK5/p25 (Supplementary Fig. S6b). Thus, the inhibitory effect of 3HB on CDK5 might merely has a limited effect on the phosphorylation of PPARγ Ser273. Additionally, 3HB increased the level of Ca^2+^ in adipocytes (Fig. 4B), which might activate calpain to cut p35 into p25, and finally offset the effect of 3HB. Nevertheless, abnormally activated CDK5 was observed in neurodegenerative diseases.^43^ 3HB could improve neurodegenerative diseases such as Alzheimer’s disease and Parkinson’s syndrome, while the current mechanism was only limited to its anti-inflammatory and antioxidant stress effects.^44–46^ For future studies, the effect of 3HB on CDK5/p35 provides a new molecular mechanism for the treatment of neurodegenerative diseases.

In conclusion, our study systematically elaborated the therapeutic effects of 3HB in T2D mice and identified a possible signaling pathway by which 3HB exerts its antidiabetic effects, providing strong evidence for its potential as a therapeutic drug in the clinical management of T2D.

## Materials and methods

### Animal treatments

Four-week-old male C57BL/6 mice were purchased from Vital River Laboratory Animal Technology Co, Beijing, China. HCAR2^-/-^ mice on C57BL/6 background were kindly donated by Dr. Wei Wang of Jilin University. 4-week-old male db/db mice on C57BL/6 background were purchased from GemPharmatech, Nanjing, China. All mice were housed in a specific pathogen-free animal facility in Tsinghua animal house, with a 12 h light and 12 h dark cycle. All animal procedures were approved by the Institutional Animal Care and Use Ethic Committee of Tsinghua University (NO. 19-CGQ1).

Except normal diet control group (ND), all the animals were fed a high-fat diet from 4-week-old. For db/db, mice were grouped based on glucose levels at an age of 5 weeks. For STZ induced T2D model, 8-week-old C57BL/6 male mice were injected intraperitoneally with STZ at the dose of 50 mg/kg for five consecutive days. From 7 weeks of age, animals were given water (control) or 10% 1,3-BDO solution as drink fluid.

### Histology analysis

Collected adipose tissue, liver and kidney were fixed in 4% paraformaldehyde for histopathological analysis. Firstly, the fixed tissues were treated with gradient dehydration, followed with paraffin embedding. The embedded tissues were cut into 5 µm sections to perform H&E staining. The H&E stained sections were visualized using an Olympus BX43.

### Intraperitoneal glucose tolerance test (IPGTT)

Mice were fasted for 12 h, and then 2 g/kg glucose was injected into the intraperitoneal cavity. Blood glucose levels were measured at 0, 30, 60, and 120 min with a glucometer (Sinocare, China).

### Biochemical measurements

Blood was collected from the heart of T2D mice under general anesthesia. The samples were centrifuged at 4 °C, 5000 × *g* for 5 min and the supernatants were collected. Fasting serum insulin was detected with an ELISA kit. Serum triglyceride, cholesterol, high-density lipoprotein cholesterol, low-density lipoprotein cholesterol, alanine aminotransferase, aspartate aminotransferase and total bilirubin were measured with a fully automatic biochemical analysis meter (Rayto Life, China). Blood 3HB concentration was detected with a blood ketone body meter (Abbott, USA)

### Cell lines and culture

3T3-L1 preadipocytes and HEK293T cells were obtained from ATCC. Cells were cultured in BMDM growth high glucose Dulbecco’s modified Eagle’s medium with 10% FBS and 100 μg/mL penicillin and streptomycin. 3T3-L1 preadipocytes were grown and induced to differentiate as described.^28^ To examine the effect on differentiation, 3T3-L1 preadipocytes were induced to differentiate in the presence of 3HB or rosiglitazone. At Day 8 post-differentiation, the cells were stained with Oil Red O. To preliminarily examine the effect on PPARγ phosphorylation at Ser273, 3T3-L1 adipocytes were pretreated with TNF-α for 1 h, followed by treatment with 3HB or rosiglitazone for another 1 h. For the measurement of PKA, Raf1, ERK1/2 and PPARγ phosphorylation, 3T3-L1 adipocytes were treated with 3HB or rosiglitazone for 1 h. To detect the effect of 3HB on PPARγ regulated downstream genes expression at protein level, 3T3-L1 adipocytes were incubated in a serum-free medium for 16 h followed by the treatment with 3HB or rosiglitazone alone, or together with inhibitors (SCH772984 or GW9662) for 48 h.

3HB was dissolved in sterile water, and other chemicals used in the treatment were dissolved in DMSO. The vehicle controls for each treatment were sterile water (3HB 0 mM).

### PPARγ luciferase reporter and ligand competitive binding assay

HEK293T cells were transfected with PPRE-TK-Luciferase reporter along with PPARγ and RXRα expression vectors. 6 h after transfection, the cells were treated with 3HB or rosiglitazone for 18 h and then harvested for the luciferase assay. Luciferase activities were normalized to Renilla activities co-transfected as an internal control. LanthaScreen^TM^ TR-FRET PPARγ competitive binding assay was performed according to the manufacturer’s instructions.

### Intracellular calcium ion, cAMP and kinase activity measurement

3T3-L1 adipocytes with or without siRNA knock down of HCAR2 were treated with 3HB or different positive control compound for 1 h. Intracellular calcium ion levels in 3HB and 1 μM ATP groups were detected with a Fura-2/AM probe kit. Intracellular cAMP concentration in 3HB and 100 μM forskolin groups were measured with a cAMP ELISA kit. ERK1/2 activity in 3HB and 10 μM SCH772984 groups were quantified with a ERK1/2 kinase Activity Quantitative detection Kit. CDK5/p35 and CDK5/p25 activity were also measured with kinase Activity Quantitative detection Kits.

### Knockdown by siRNA

Mature 3T3-L1 adipocytes were cultured in high glucose DMEM with 10% FBS and 100 μg/mL penicillin and streptomycin. siRNA was transfected into 3T3-L1 adipocytes with Lipofectamine 3000. Gene expression of *Hcar2* was detected after 24 h. Other in vitro studies were performed after 48 h of transfection.

### Glucose uptake assay

3T3-L1 adipocytes with or without HCAR2 knockdown by siRNA were treated with 3HB or rosiglitazone for 24 h. Glucose uptake was measured by a bioluminescent assay using the Glucose Uptake-Glo^TM^ Assay Kit.

### Western blot analysis

The adipose tissue from T2D mice or 3T3-L1 adipocytes were lysed in cell lysis buffer (Yeasen, China) with phosphatase inhibitor cocktail, protease inhibitor and PMSF. Western blot analysis were performed with 30–50 μg protein using commercially available or donated antibodies to the following: pPKA (Thr197), PKA, pRaf1 (Ser259), Raf1, pERK1/2 (Thr202/Tyr204), ERK1/2, PPARγ, GLUT4 and GAPDH (Cell Signaling), pPPARγ(Ser273) (Bioss, China), HCAR2 (ABclonal, China), and adiponectin (donated by Dr. Li Zhen of Tsinghua University). Secondary antibodies were obtained from Cell Signaling.

### Isolation of RNA and analysis by real-time quantitative PCR

Total RNA of adipose tissue or 3T3-L1 adipocytes was extracted with TRIzol reagent and the cDNAs were generated by reverse transcription kit. Real-Time Quantitative PCR (qPCR) was performed with the standard protocols in ABI 7500 Fast Real-Time PCR System (Applied Biosystems™ 7500, ThermoFisher, USA) using SYBR Green Master Mix. GAPDH gene expression was used to normalize the expression of mRNA for genes of interest. Primers used for qPCR are listed (Table 1).

**Table 1:** Primers of qPCR

Genes	Forward primers	Reverse primers
*HCAR2*	5′-TCCAAGTCTCCAAAGGTGGT-3′	5′-TGTTTCTCTCCAGCACTGAGTT-3′
*aP2*	5′-AAGGTGAAGAGCATCATAACCCT-3′	5′-TCACGCCTTTCATAACACATTCC-3′
*Adipsin*	5′-CATGCTCGGCCCTACATGG-3′	5′-CACAGAGTCGTCATCCGTCAC-3′
*LPL*	5′-GGGAGTTTGGCTCCAGAGTTT-3′	5′-TGTGTCTTCAGGGGTCCTTAG-3′
*Glut4*	5′-GTGACTGGAACACTGGTCCTA-3′	5′-CCAGCCACGTTGCATTGTAG-3′
*Adiponectin*	5′-TGTTCCTCTTAATCCTGCCCA-3′	5′-CCAACCTGCACAAGTTCCCTT-3′
*CD36*	5′-AAGCTATTGCGACATGATT-3′	5′-GATCCGAACACAGCGTAGAT-3′
*Resistin*	5′-AAGAACCTTTCATTTCCCCTCCT-3′	5′-GTCCAGCAATTTAAGCCAATGTT-3′
*Leptin*	5′-GAGACCCCTGTGTCGGTTC-3′	5′-CTGCGTGTGTGAAATGTCATTG-3′
*PPARγ*	5′-GCATGGTGCCTTCGCTGA-3′	5′-TGGCATCTCTGTGTCAACCATG-3′
*Car3*	5′-TGACAGGTCTATGCTGAGGGG-3′	5′-CAGCGTATTTTACTCCGTCCAC-3′
*Nr1d1*	5′-TACATTGGCTCTAGTGGCTCC-3′	5′-CAGTAGGTGATGGTGGGAAGTA-3′
*Nr1d2*	5′-TGAACGCAGGAGGTGTGATTG-3′	5′-GAGGACTGGAAGCTATTCTCAGA-3′
*GAPDH*	5′-AGAACATCATCCCTGCATCC-3′	5′-TCCACCACCCTGTTGCTGTA-3′

### Transcriptome sequencing and analysis

Total RNA of adipose tissue was collected to analyze the transcriptome. A cDNA library was constructed using qualified adipose tissue RNA samples. The transcriptome sequencing processes of adipose tissue were performed by Novogene (Beijing, China) using Illumina HiSeqTM 4000 sequencer. The adaptors of paired-end reads were trimmed and quality control checks were carried out using trim-galore (v.0.6.0). Reads were aligned to mouse genome reference (GRCm38.p6) from GENCODE using STAR (v.2.7.3a). FeatureCounts (v.1.6.3) counted reads stored in BAM format to exon sites of genes included in GTF files from GENCODE. Differential gene expression analysis was conducted using raw counts as input using R (v.3.5.1) package DESeq2 (v.1.22.2). Kyoto Encyclopedia of Genes and Genomes (KEGG) analysis and Gene ontology (GO) enrichment analysis of differential genes were performed by R (v.3.5.1) package clusterProfiler (v3.10.1).

### Quantification and statistical analysis

Results are presented as the mean ± standard deviation (SD). The statistical differences between two groups were analyzed using the two-tailed *t*-test or one-way ANOVA by GraphPad Prism software 9. The data were considered significant difference as the *p*-value < 0.05.

## Supplementary information

Supplementary Materials for 3-Hydroxybutyrate Ameliorates Insulin Resistance by Inhibiting PPARγ Ser273 Phosphorylation in Type 2 Diabetic Mice

## Data Availability

All data needed to evaluate the conclusions in the paper are present in the paper and its supplementary information files. Requests for resources and further information should be directed to the lead contact, Guo-Qiang Chen (chengq@mail.tsinghua.edu.cn). Source transcriptomic data are available through https://www.ncbi.nlm.nih.gov/bioproject/847336. Accession number: PRJNA847336.

## References

[1] Magliano DJ, E. J. B. & International Diabetes Federation. *IDF Diabetes Atlas* 10th edn (International Diabetes Federation, 2021).

[2] Tontonoz P, Spiegelman BM (2008). Fat and beyond: the diverse biology of PPARgamma. Annu. Rev. Biochem..

[3] Hameed M, Khan K, Salman S, Mehmood N (2017). Dose comparison and side effect profile of metformin extended release versus metformin immediate release. J. Ayub. Med. Coll. Abbottabad..

[4] Rizos CV, Elisaf MS, Mikhailidis DP, Liberopoulos EN (2009). How safe is the use of thiazolidinediones in clinical practice?. Expert Opin. Drug Saf.

[5] McGovern A (2018). Comparison of medication adherence and persistence in type 2 diabetes: a systematic review and meta-analysis. Diabetes Obes. Metab..

[6] Brunmeir R, Xu F (2018). Functional regulation of PPARs through post-translational modifications. Int. J. Mol. Sci..

[7] Choi JH (2011). Antidiabetic actions of a non-agonist PPARgamma ligand blocking Cdk5-mediated phosphorylation. Nature.

[8] Banks AS (2015). An ERK/Cdk5 axis controls the diabetogenic actions of PPARgamma. Nature.

[9] Newman JC, Verdin E (2017). beta-hydroxybutyrate: a signaling metabolite. Annu. Rev. Nutr..

[10] Layden BT, Angueira AR, Brodsky M, Durai V, Lowe WL (2013). Short chain fatty acids and their receptors: new metabolic targets. Transl. Res..

[11] Soga T (2003). Molecular identification of nicotinic acid receptor. Biochem. Biophys. Res. Commun..

[12] Exton JH (1996). Regulation of phosphoinositide phospholipases by hormones, neurotransmitters, and other agonists linked to G proteins. Annu. Rev. Pharmacol. Toxicol..

[13] Gaidarov I (2013). Differential tissue and ligand-dependent signaling of GPR109A receptor: implications for anti-atherosclerotic therapeutic potential. Cell Signal.

[14] Chen Y (2018). beta-Hydroxybutyrate protects from alcohol-induced liver injury via a Hcar2-cAMP dependent pathway. J. Hepatol..

[15] Soto-Mota A, Norwitz NG, Evans R, Clarke K, Barber TM (2021). Exogenous ketosis in patients with type 2 diabetes: Safety, tolerability and effect on glycaemic control. Endocrinol. Diabetes Metab..

[16] Furman BL (2021). Streptozotocin-induced diabetic models in mice and rats. Curr. Protoc..

[17] Miller SA, Dymsza HA (1967). Utilization by the rat of 1,3-butanediol as a synthetic source of dietary energy. J. Nutr.

[18] Desrochers S, David F, Garneau M, Jette M, Brunengraber H (1992). Metabolism of R- and S-1,3-butanediol in perfused livers from meal-fed and starved rats. Biochem. J..

[19] Youm YH (2015). The ketone metabolite beta-hydroxybutyrate blocks NLRP3 inflammasome-mediated inflammatory disease. Nat. Med..

[20] Chakraborty S (2018). Salt-responsive metabolite, beta-hydroxybutyrate, attenuates hypertension. Cell. Rep..

[21] Jadeja RN (2019). Loss of GPR109A/HCAR2 induces aging-associated hepatic steatosis. Aging (Albany NY).

[22] Chawla A, Schwarz EJ, Dimaculangan DD, Lazar MA (1994). Peroxisome proliferator-activated receptor (PPAR) gamma: adipose-predominant expression and induction early in adipocyte differentiation. Endocrinology.

[23] Semple RK, Chatterjee VK, O’Rahilly S (2006). PPAR gamma and human metabolic disease. J. Clin. Invest.

[24] Kroker AJ, Bruning JB (2015). Review of the structural and dynamic mechanisms of PPARgamma partial agonism. PPAR Res..

[25] Schiel R, Beltschikow W, Steiner T, Stein G (2006). Diabetes, insulin, and risk of cancer. Methods Find Exp. Clin. Pharmacol..

[26] Zhang Y (2020). WSF-7 inhibits obesity-mediated PPARgamma phosphorylation and improves insulin sensitivity in 3T3-L1 adipocytes. Biol. Pharm. Bull..

[27] Choi SH, Chung SS, Park KS (2018). Re-highlighting the action of PPARgamma in treating metabolic diseases. F1000Res.

[28] Choi JH (2010). Anti-diabetic drugs inhibit obesity-linked phosphorylation of PPARgamma by Cdk5. Nature.

[29] Hu E (2018). Beta-hydroxybutyrate promotes the expression of BDNF in hippocampal neurons under adequate glucose supply. Neuroscience.

[30] Dumaz N, Marais R (2003). Protein kinase A blocks Raf-1 activity by stimulating 14-3-3 binding and blocking Raf-1 interaction with Ras. J. Biol. Chem..

[31] Gerits N, Kostenko S, Shiryaev A, Johannessen M, Moens U (2008). Relations between the mitogen-activated protein kinase and the cAMP-dependent protein kinase pathways: comradeship and hostility. Cell. Signal..

[32] Pabbidi MR (2016). Inhibition of cAMP-dependent PKA activates beta2-adrenergic receptor stimulation of cytosolic phospholipase A2 via Raf-1/MEK/ERK and IP3-dependent Ca2+ signaling in atrial myocytes. PLoS One.

[33] Cahill GF (2006). Fuel metabolism in starvation. Annu. Rev. Nutr..

[34] Kitabchi AE, Umpierrez GE, Miles JM, Fisher JN (2009). Hyperglycemic crises in adult patients with diabetes. Diabetes Care.

[35] Puchalska P, Crawford PA (2017). Multi-dimensional roles of ketone bodies in fuel metabolism, signaling, and therapeutics. Cell. Metab..

[36] Li Z (2021). Applications and mechanism of 3-Hydroxybutyrate (3HB) for prevention of colonic inflammation and carcinogenesis as a food supplement. Mol. Nutr. Food. Res..

[37] Zhang SJ (2021). Ketone body 3-Hydroxybutyrate ameliorates atherosclerosis via receptor Gpr109a-mediated calcium influx. Adv. Sci. (Weinh).

[38] Nielsen R (2019). Cardiovascular effects of treatment with the ketone body 3-hydroxybutyrate in chronic heart failure patients. Circulation.

[39] Gambhir D (2012). GPR109A as an anti-inflammatory receptor in retinal pigment epithelial cells and its relevance to diabetic retinopathy. Invest. Ophthalmol. Vis. Sci..

[40] Meex RCR, Blaak EE, van Loon LJC (2019). Lipotoxicity plays a key role in the development of both insulin resistance and muscle atrophy in patients with type 2 diabetes. Obes. Rev..

[41] Takahashi Y (2019). Effects of beta-hydroxybutyrate treatment on glycogen repletion and its related signaling cascades in epitrochlearis muscle during 120 min of postexercise recovery. Appl. Physiol. Nutr. Metab..

[42] Chen J (2022). Mechanism of reduced muscle atrophy via ketone body (D)-3-hydroxybutyrate. Cell. Biosci.

[43] Pao PC, Tsai LH (2021). Three decades of Cdk5. J. Biomed. Sci..

[44] Zhang J (2013). 3-Hydroxybutyrate methyl ester as a potential drug against Alzheimer’s disease via mitochondria protection mechanism. Biomaterials.

[45] Wu Y (2020). BHBA treatment improves cognitive function by targeting pleiotropic mechanisms in transgenic mouse model of Alzheimer’s disease. FASEB J.

[46] Fu SP (2015). Anti-inflammatory effects of BHBA in both in vivo and in vitro Parkinson’s disease models are mediated by GPR109A-dependent mechanisms. J Neuroinflammation.

