# Supplementary material for: 3-Hydroxybutyrate ameliorates insulin resistance by inhibiting PPARγ Ser273 phosphorylation in type 2 diabetic mice
Source: Signal Transduct Target Ther. 2023 May 26;8:190. doi: 10.1038/s41392-023-01415-6 (PMC10212965; doi:10.1038/s41392-023-01415-6)

Supplementary Materials for

3-Hydroxybutyrate Ameliorates Insulin Resistance by Inhibiting PPARγ Ser273 Phosphorylation in Type 2 Diabetic Mice

Yudian Zhang, Zihua Li, Xinyi Liu, Xinyu Chen, Shujie Zhang, Yuemeng Chen, Jiangnan Chen, Jin Chen, Fuqing Wu, Guo-Qiang Chen

Correspondence to: chengq@mail.tsinghua.edu.cn

**This PDF file includes:**

Figures. S1 to S6

Table S1.

Unedited Gels

Figure. S1.


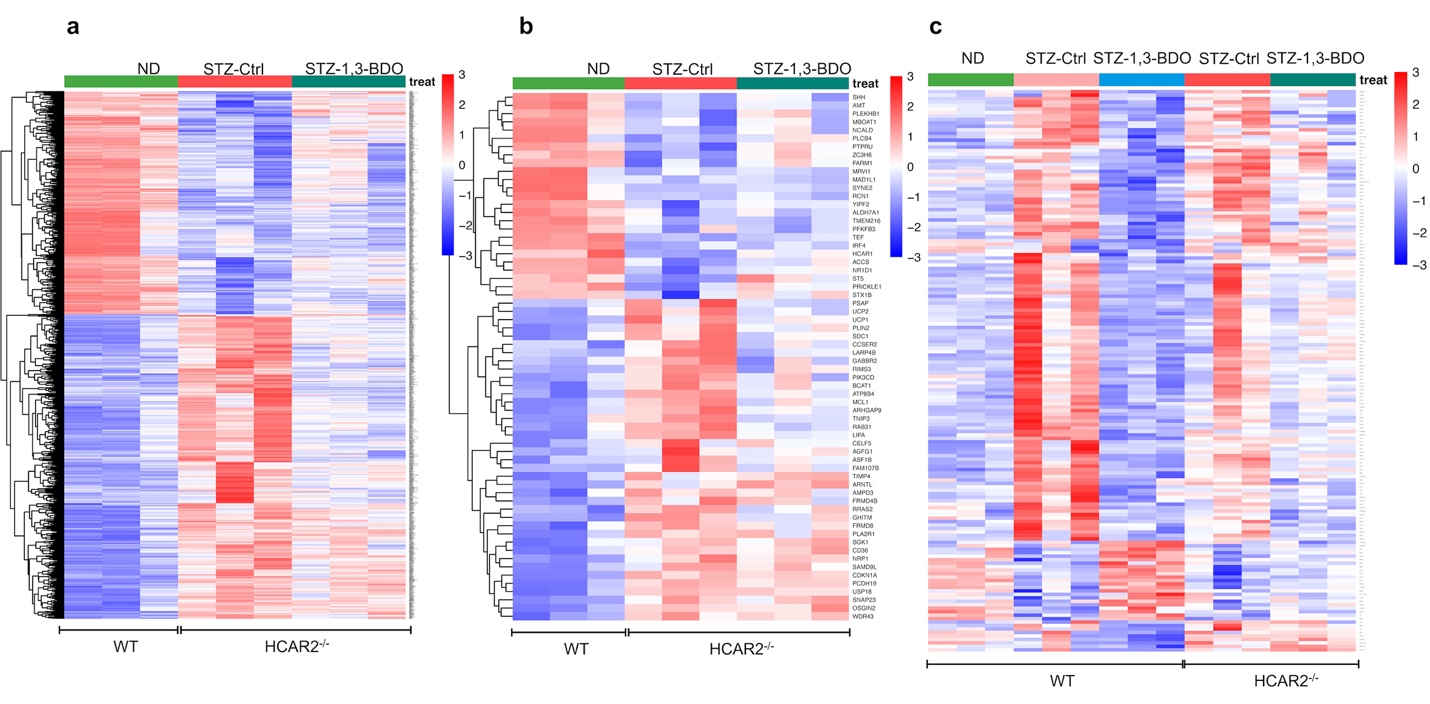


**Fig. S1. Transcriptomics Analysis for Adipose Tissue of STZ Induced HCAR2^-/-^ Type 2 Diabetic Mice.** (a) After 8 weeks of indicated treatment, the adipose tissue of Normal diet mice (ND) and STZ induced HCAR2^-/-^ type 2 diabetic mice was collected and conducted the differential gene expression analysis (*p*＜0.05) by RNA sequencing. (b) PPARγ regulated genes in differentially expressed genes of STZ HCAR2^-/-^ mice. (c). Differentially expressed genes of all STZ induced type 2 diabetic groups.

Figure. S2.


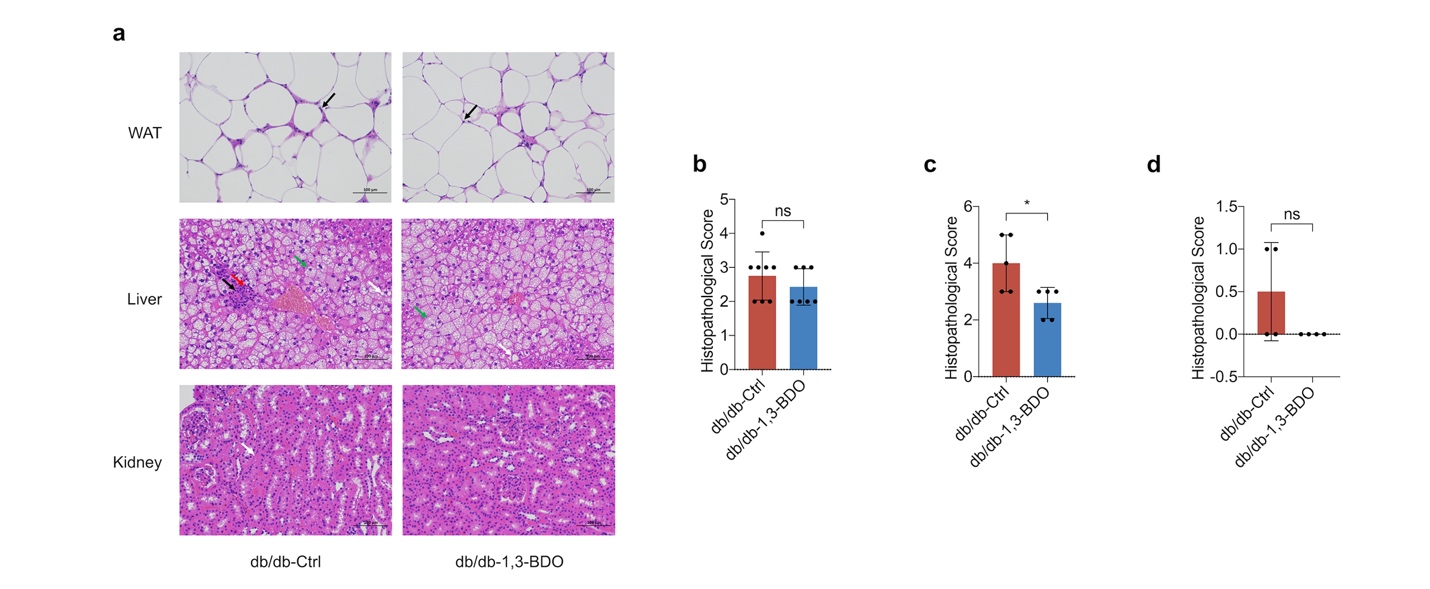


**Fig. S2. 1,3-BDO Treatment Reduces Tissue Injury in db/db Mice.** (a) Representative images of hematoxylin and eosin staining of adipose tissue, liver and kidney sections of mice with indicated treatment. (b-d) Pathological damage score of adipose tissue, liver and kidney, respectively. The different colored arrows represent: red, necrocytosis; black, lymphocytes infiltration; white, fatty degeneration; blue, hydropic degeneration; green, cytoplasmic vacuolation; yellow, abnormal karyotype; orange, abnormal volume of cells; purple, perivascular hemorrhage. ﻿Scale bar: 100 μm.

Figure. S3.


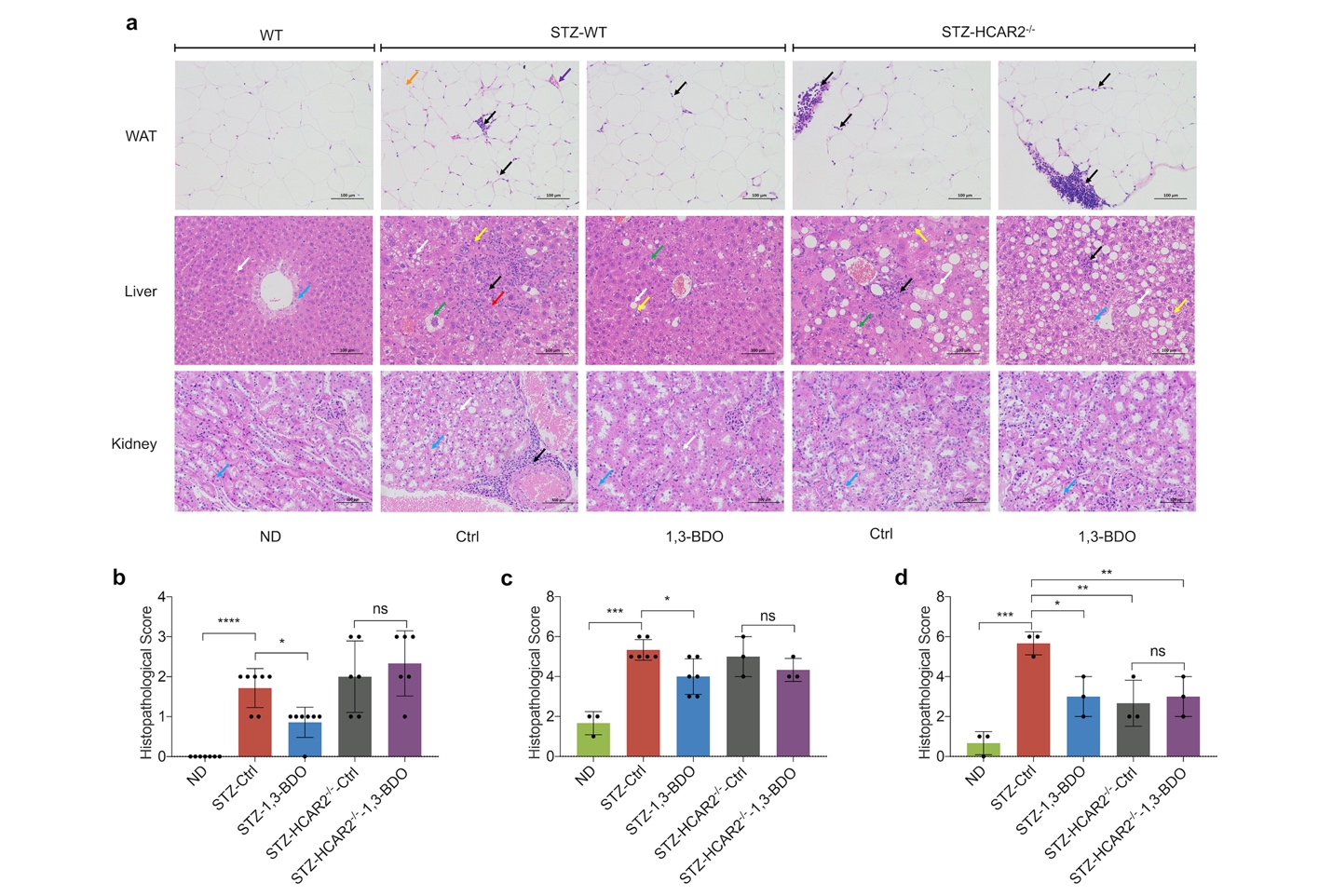


**Fig. S3. 1,3-BDO Treatment Reduces Tissue Injury in STZ Induced Type 2 Diabetic Mice.** (a) Representative images of hematoxylin and eosin staining of adipose tissue, liver and kidney sections of mice with indicated treatment. (b-d) Pathological damage score of adipose tissue, liver and kidney, respectively. n=6. Data reported as mean ± SD, * *p*＜0.05, ** *p*＜0.001, and *** *p*＜0.0001, **** *p*＜0.00001. Scale bar: 100 μm.

Figure. S4.


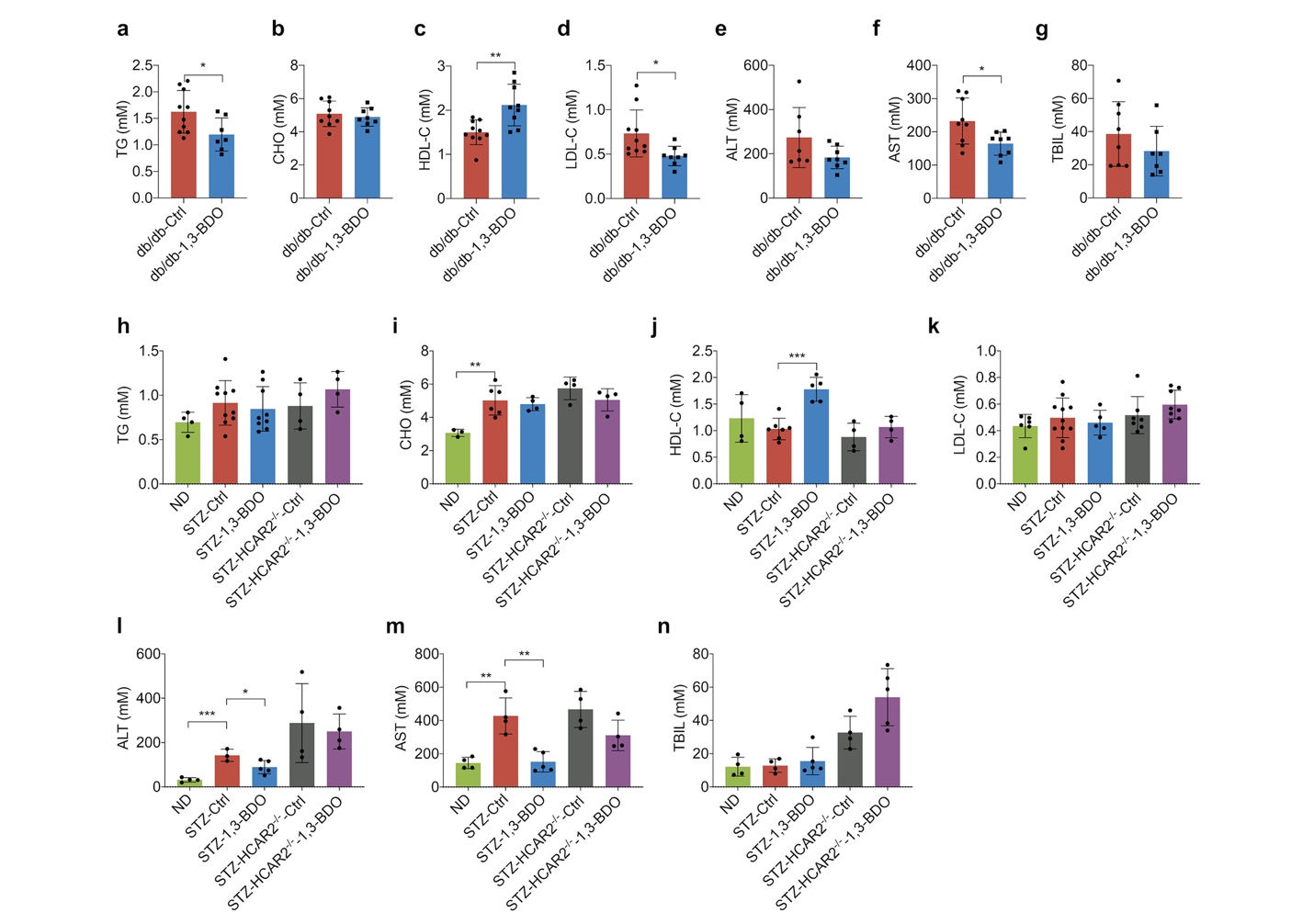


**Fig. S4. 1,3-BDO Treatment Improves Serum Lipids and Liver Function of Type 2 Diabetic Mice.** (a-g) Serum triglyceride (TG), cholesterol (CHO), high-density lipoprotein cholesterol (HDL-C), low-density lipoprotein cholesterol (LDL-C), alanine aminotransferase (ALT), aspartate aminotransferase (AST) and total bilirubin (TBIL) of db/db mice with indicated treatment. (h-n) Serum triglyceride (TG), cholesterol (CHO), high-density lipoprotein cholesterol (HDL-C), low-density lipoprotein cholesterol (LDL-C), alanine aminotransferase (ALT), aspartate aminotransferase (AST) and total bilirubin (TBIL) of STZ induced type 2 diabetic mice with indicated treatment. n=6. Data reported as mean ± SD, * *p*＜0.05, ** *p*＜0.001, and *** *p*＜0.0001.

Figure. S5.


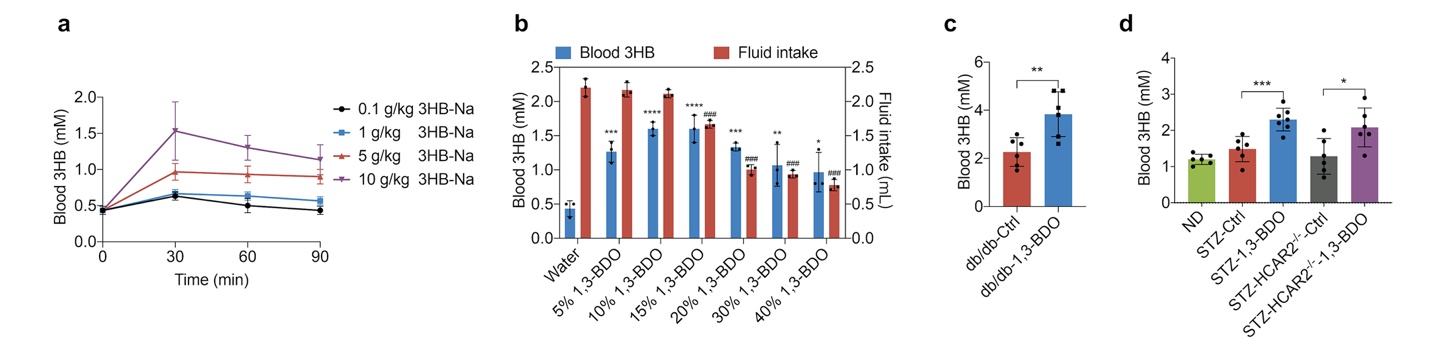


**Fig. S5. Blood 3HB Level in Mice with Different Ketone Body Supplements.** (a) 8-week-old male C57BL/6J mice were treated with different dose of 3HBNa by oral gavage. Blood 3HB concentrations were measured after 0, 30, 60, and 90 min of the treatment. (b) 8-week-old male C57BL/6J mice were given water or different concentration 1,3-butanediol after 24 hours water deprivation. Blood 3HB concentrations were measured after 2 hour of fluid intake. (c) Fasting blood 3HB levels of 12-week-old db/db type 2 diabetic mice treated with or without 10% 1,3-BDO. (d) Fasting blood 3HB levels of 12-week-old STZ induced type 2 diabetic mice with or without 10% 1,3-BDO. n=6-7. Data reported as mean ± SD, * *p*＜0.05, ** *p*＜0.001, and *** *p*＜0.0001, **** *p*＜0.00001. ### *p*＜0.0001 for fluid intake.

Figure. S6.


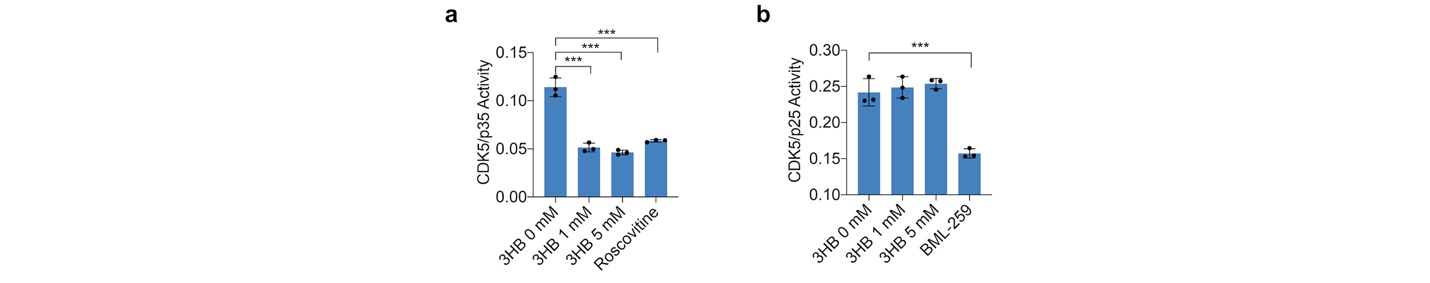


**Fig. S6. Effect of 3HB on CDK5 Activity.** The kinase activity of CDK5/p35 (a) or CDK5/p25 (b) of 3T3-L1 adipocytes treated with 3HB or 10 μM roscovitine or BML-259 for 1 hour was measured using a CDK5 kinase Activity Quantitative detection Kit. Data reported as mean ± SD, *** *p*＜0.0001.

Table S1.

Regents or resources list

| Regent or resource | Source | Identifier |
| --- | --- | --- |
| Antibodies |  |  |
| ﻿Anti-rabbit IgG, HRP-linked Antibody | ﻿Cell Signaling | ﻿Cat# 7074 |
| ﻿Anti-mouse IgG, HRP-linked Antibody | Cell Signaling | ﻿Cat# 7076 |
| Anti-GAPDH | Cell Signaling | Cat# 97166S |
| Anti-GLUT4 | Cell Signaling | Cat# 2213S |
| Anti-ERK1/2 | Cell Signaling | Cat# 9194S |
| Anti-p-ERK1/2 (Thr202/Tyr204) | Cell Signaling | Cat# 9101S |
| Anti-PKA | Cell Signaling | Cat# 4782S |
| Anti-p-PKA (Thr197) | Cell Signaling | Cat# 5661S |
| Anti-Raf1 | Cell Signaling | Cat# 9422S |
| Anti-p-Raf1 (Ser259) | Cell Signaling | Cat# 9421S |
| Anti-PPARγ | Cell Signaling | Cat# 2443S |
| Anti-p-PPARγ (Ser273) | Bioss | Cat# bs-4888R |
| Anti-HCAR2 | ABclonal | Cat# A15611 |
| Anti-Adiponectin | Dr. Zhen Li at Tsinghua University | N/A |
| Chemicals and recombinant proteins | | |
| DMEM, high glucose | GIBCO | Cat# C11995500BT |
| DMEM, no glucose | GIBCO | Cat# 11966025 |
| OPTI-MEM | Thermo fisher | Cat# 31985070 |
| Fetal bovine serum | GIBCO | Cat# 10099141C |
| Trypsin | GIBCO | Cat# 25200056 |
| Penicillin & streptomycin | Hyclone | Cat# SV30010 |
| TRIzol reagent | Invitrogen | Cat# 15596018 |
| Lipofectamine 3000 | Thermo fisher | Cat# L3000015 |
| ﻿Reverse transcription kit | GenStar | Cat# A221 |
| SYBR Green Master qPCR Mix | GenStar | Cat# A301 |
| TNFα | PeproTech | Cat# 315-01A-20 |
| 3-Hydroxybutyrate | Sigma | Cat# 298360 |
| Rosiglitazone | Sigma | Cat# R2408 |
| SCH772984 | TargetMol | Cat# T6066 |
| GW9662 | Sigma | Cat# M6191 |
| 3-Isobutyl-1-methylxanthine | Sigma | Cat# I7018 |
| Dexamethasone | Sigma | Cat# D4902 |
| Insulin | Sigma | Cat# I5500 |
| Oil-red O | Sigma | Cat# O0625 |
| Streptozocin | Sigma | Cat# 309443 |
| 1,3-Butanediol | Sigma | Cat# 309443 |
| Pentobarbital sodium | Sigma | Cat# P3761 |
| Forskolin | Sigma | Cat# F3917 |
| ATP | Macklin | Cat# A832633 |
| DMSO | Beyotime | Cat# ST038 |
| Phosphatase inhibitor cocktail | Beyotime | Cat# P1045 |
| Protease inhibitor | Beyotime | Cat# P1005 |
| PMSF | Beyotime | Cat# ST506 |
| Glucose Uptake-Glo^TM^ Assay Kit | Promega | Cat# J1341 |
| Mouse Insulin ELISA kit | ALPCO | Cat# 80-INSMSU-E01 |
| cAMP Assay Kit | Nanjing Jiancheng | Cat# H164-48 |
| Fura-2/AM probe kit | AAT Bioquest | Cat# 36320 |
| ERK1/2 kinase Activity Quantitative detection Kit | Genmed | Cat# GMS50056.1 |
| CDK5/p35 kinase Activity Quantitative detection Kit | Genmed | Cat# GMS50151.1 |
| CDK5/p25 kinase Activity Quantitative detection Kit | Genmed | Cat# GMS50150.1 |
| LanthaScreen^TM^ TR-FRET PPARγ competitive binding assay kit | Invitrogen | Cat# PV4894 |
| Luciferase Reporter Gene Assay Kit | Yeasen | Cat# 11401ES60 |
| ﻿Experimental animals and cells | | |
| C57BL/6J mice | Vital River | N/A |
| HCAR2 KO mice (HCAR2^-/-^) | Dr. Wei Wang at Jilin University | N/A |
| Leptin receptor KO mice (db/db) | GemPharmatech | N/A |
| HEK293T | ATCC | N/A |
| 3T3-L1 preadipocytes | ATCC | N/A |
| Oligonucleotides | | |
| ﻿For a full list of qRT-PCR primers | ﻿Table 1 | N/A |
| siRNA for HCAR2 Forward | 5’-GCAGCUUCAGCAUCUGUUATT-3’ | |
| siRNA for HCAR2 Reverse | 5’-UAACAGAUGCUGAAGCUGCTT-3’ | |
| siRNA for Control Forward | 5’-AGAAGUGUGUGAGAAGUUCTT-3’ | |
| siRNA for Control Reverse | 5’-GAACUUCUCACACACUUCUTT-3’ | |
| ﻿Software and algorithms | | |
| ﻿GraphPad Prism | GraphPad | ﻿https://www.graphpad.com |
| ﻿Image Lab | BIO-RAD | https://commerce.bio-rad.com |
| Other | | |
| High-fat diet | SYSEBIO | Cat# D12492 |

Unedited Gels


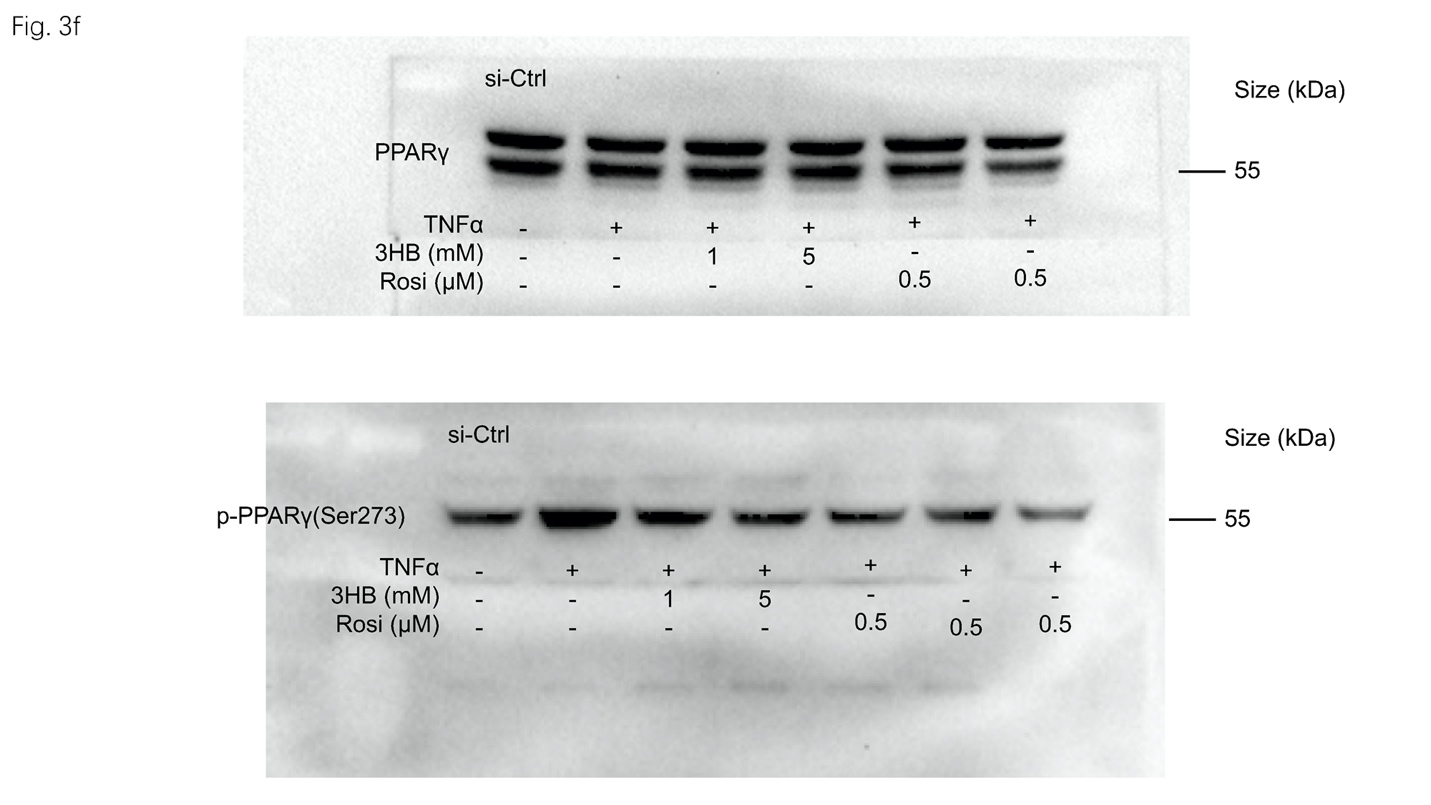


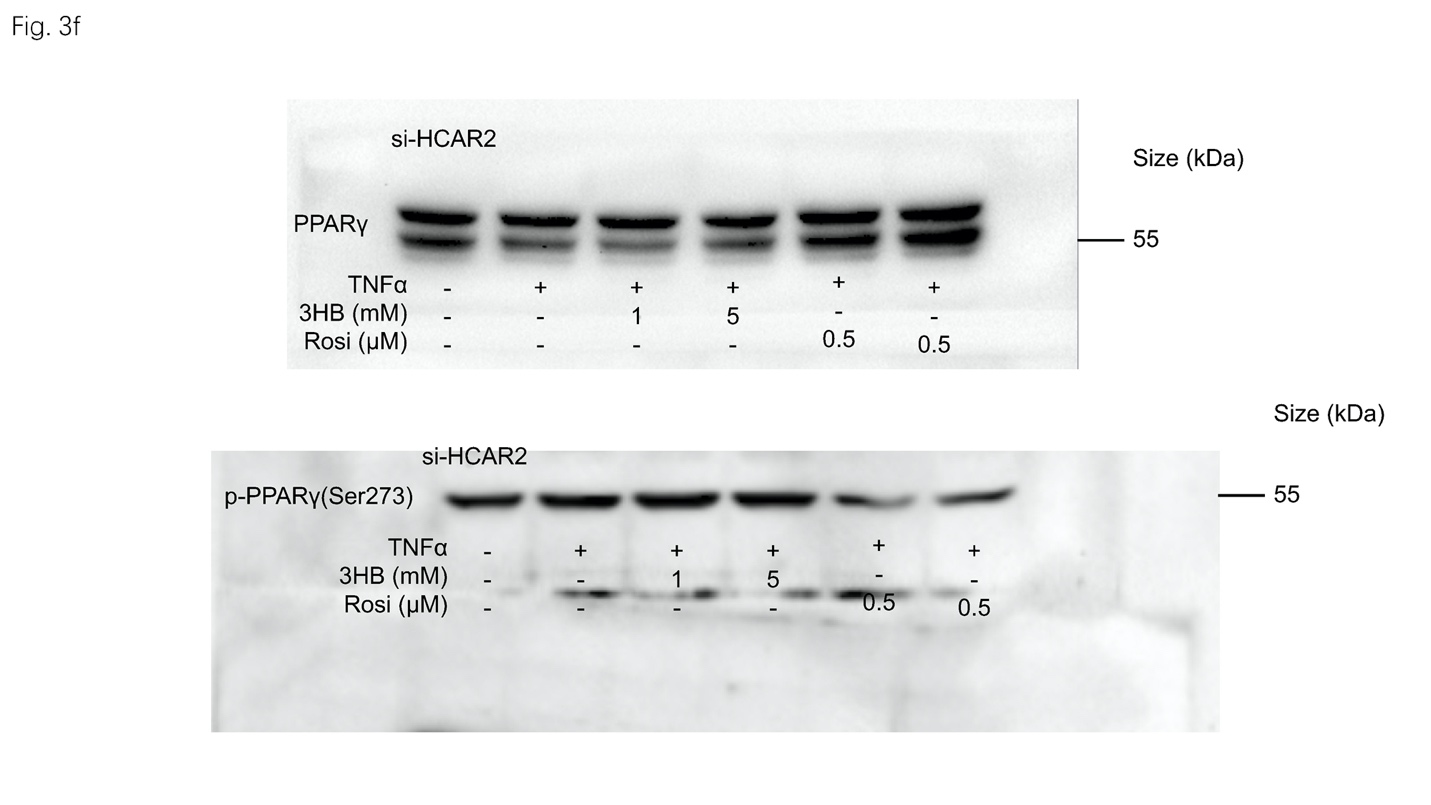


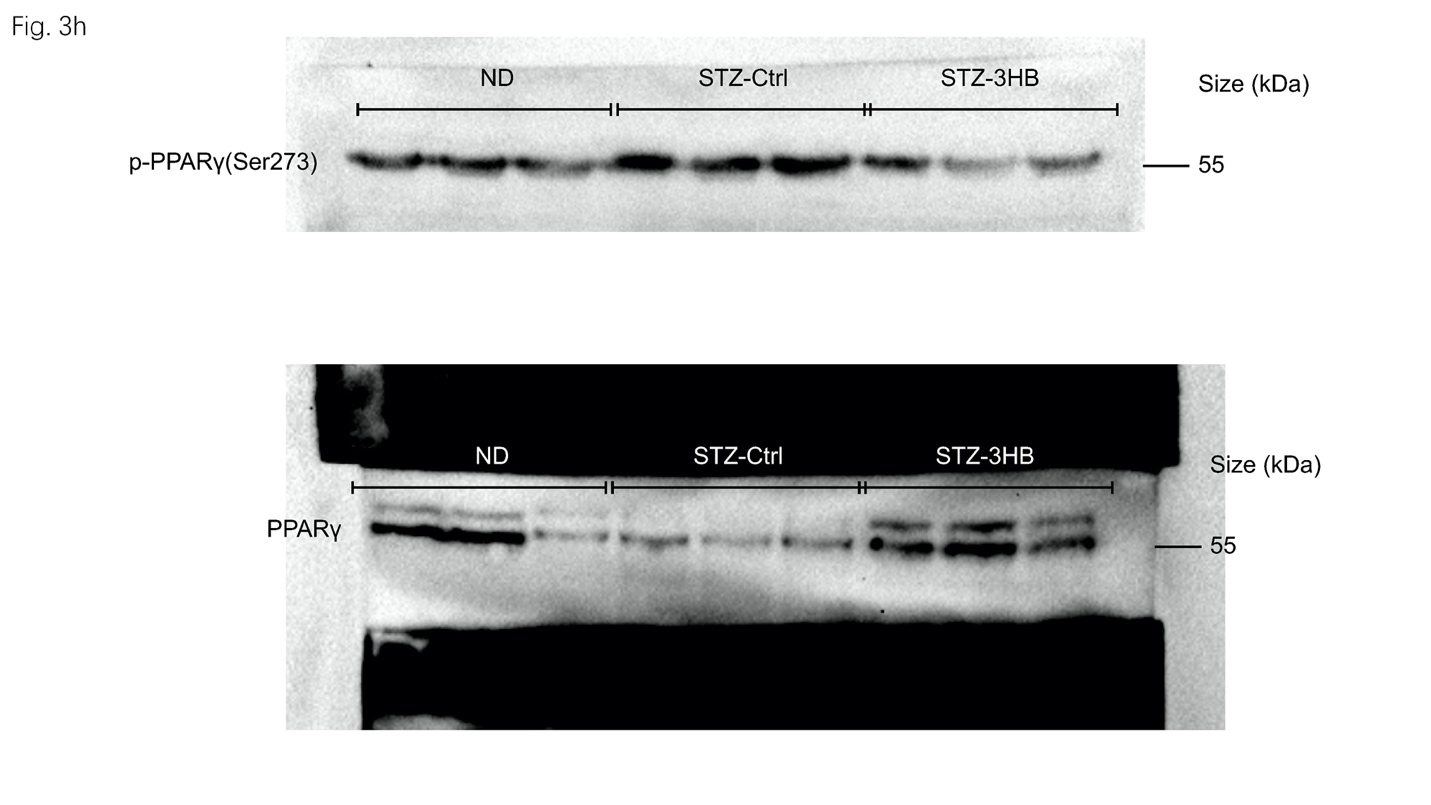

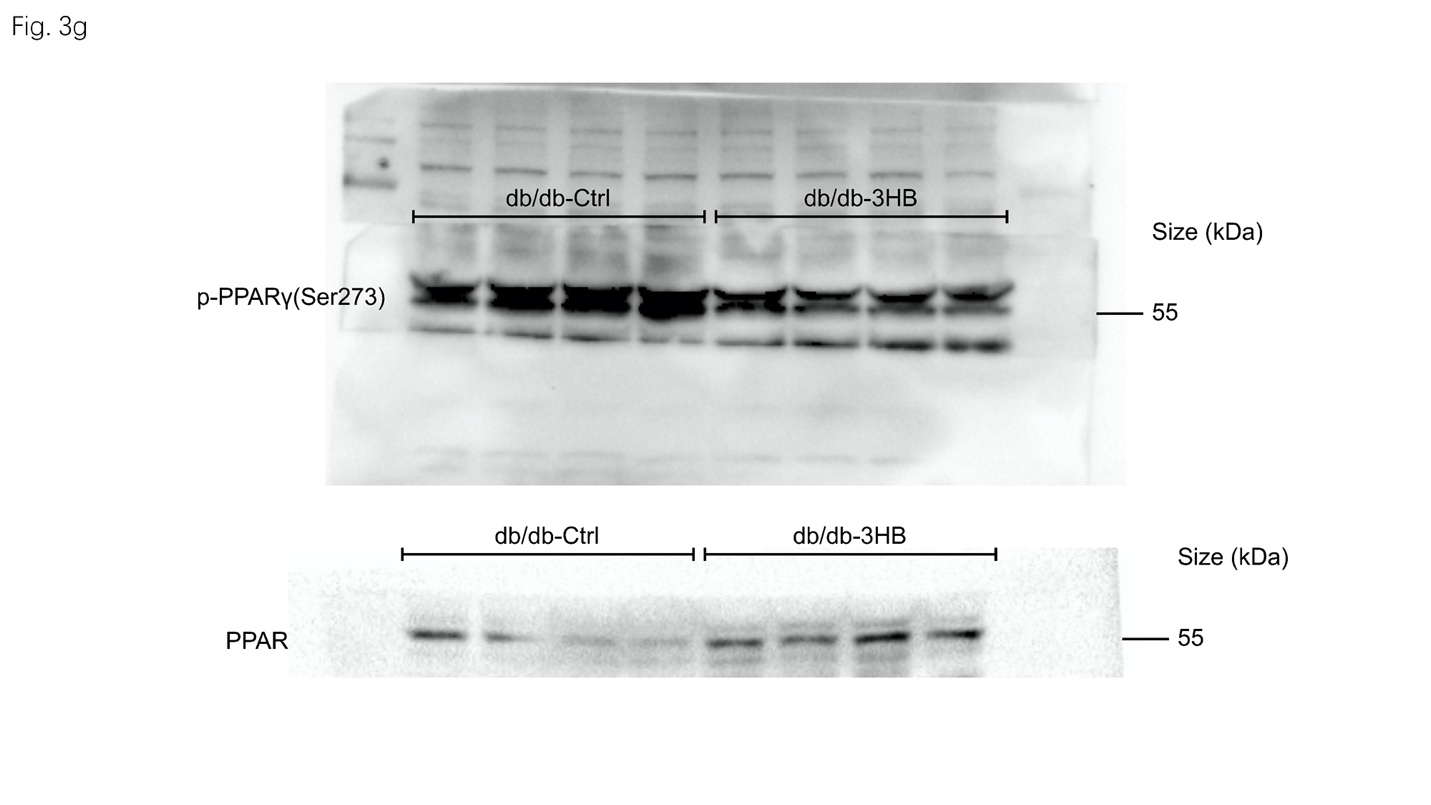


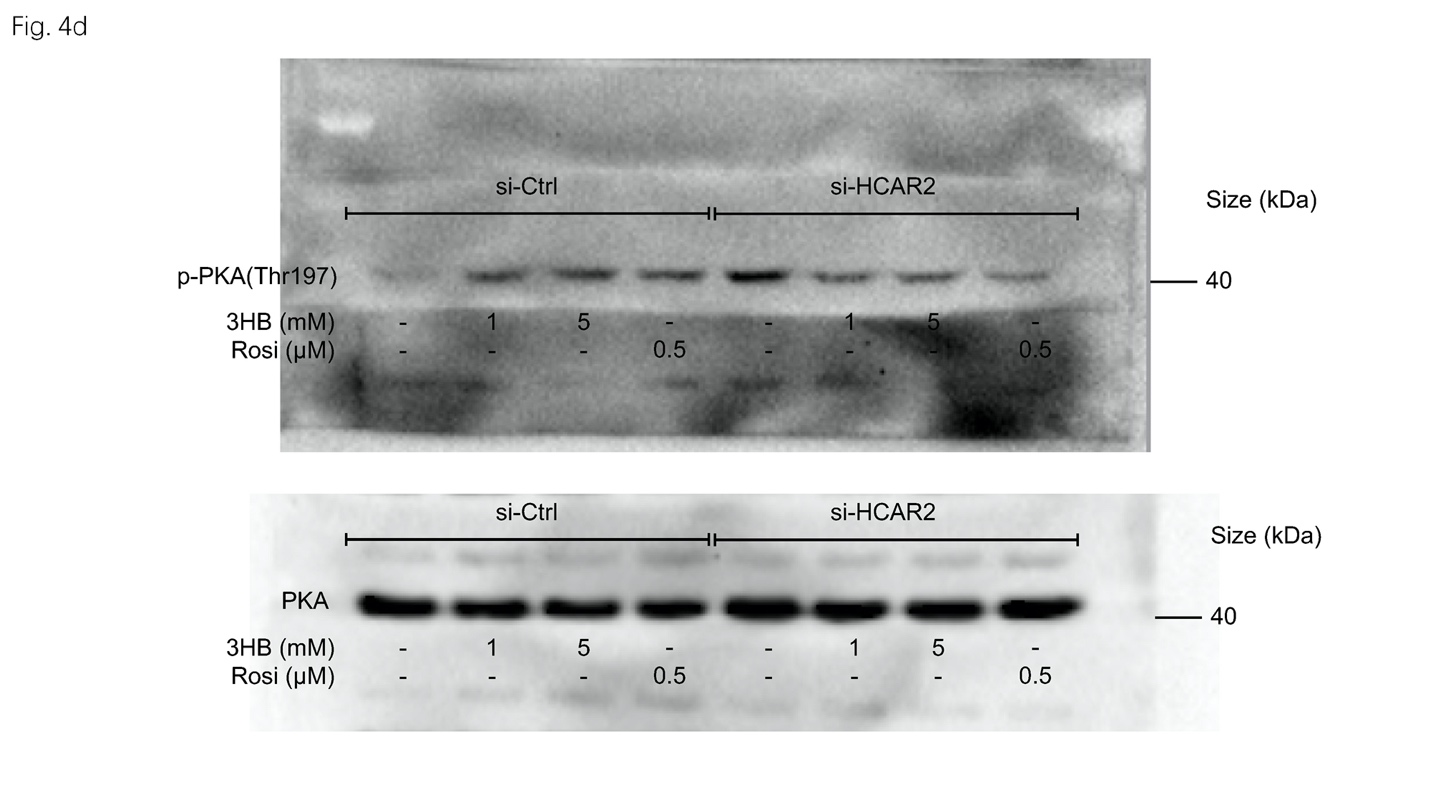

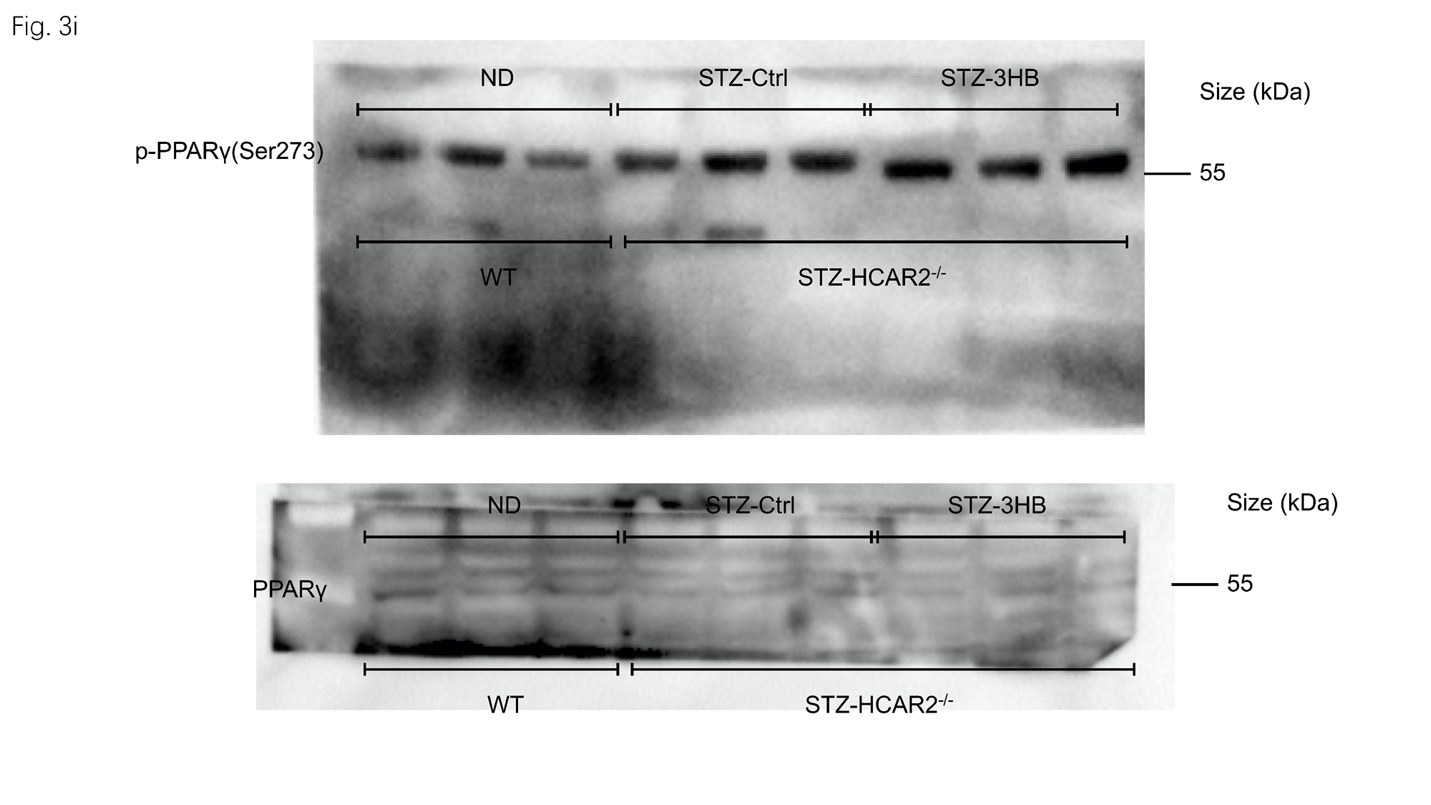


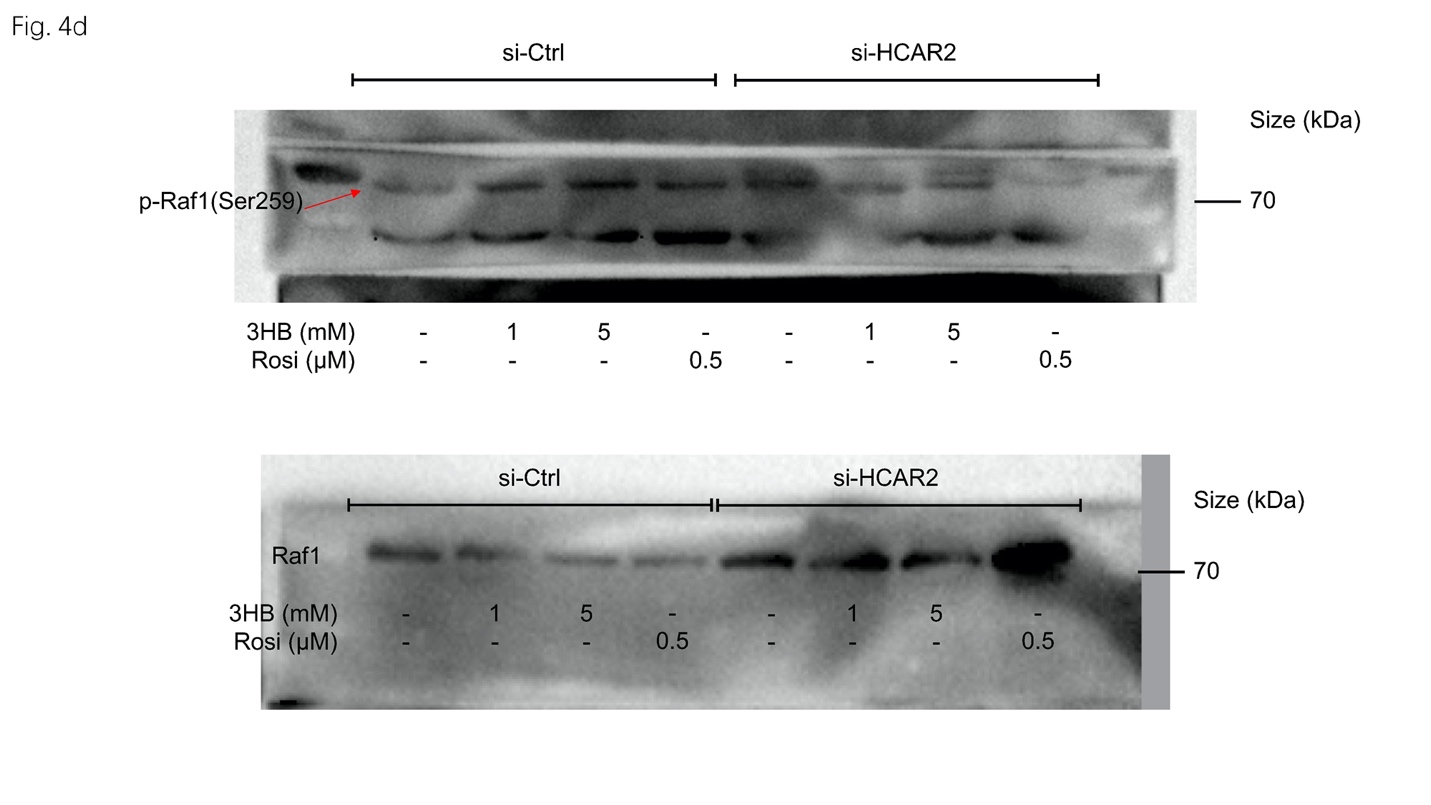


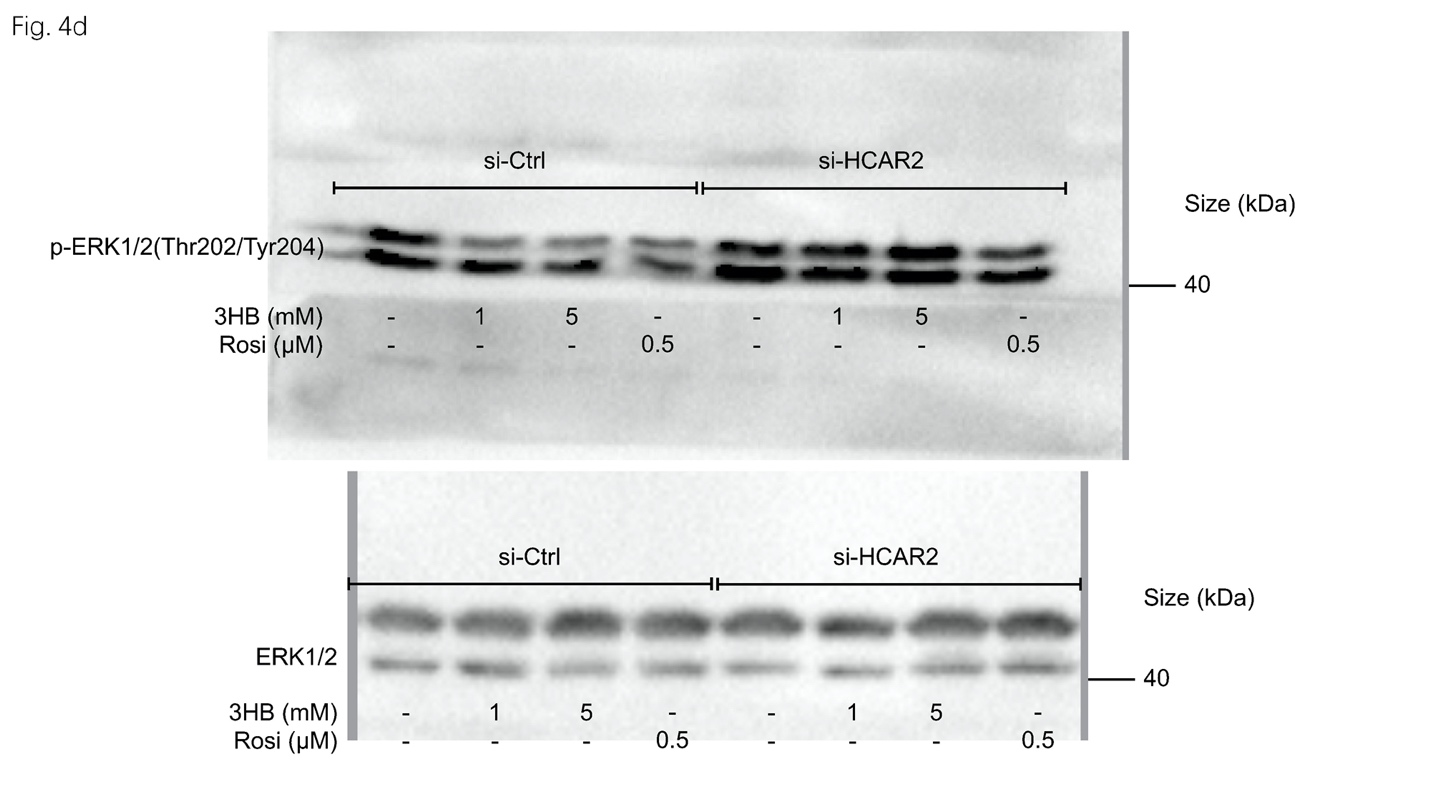


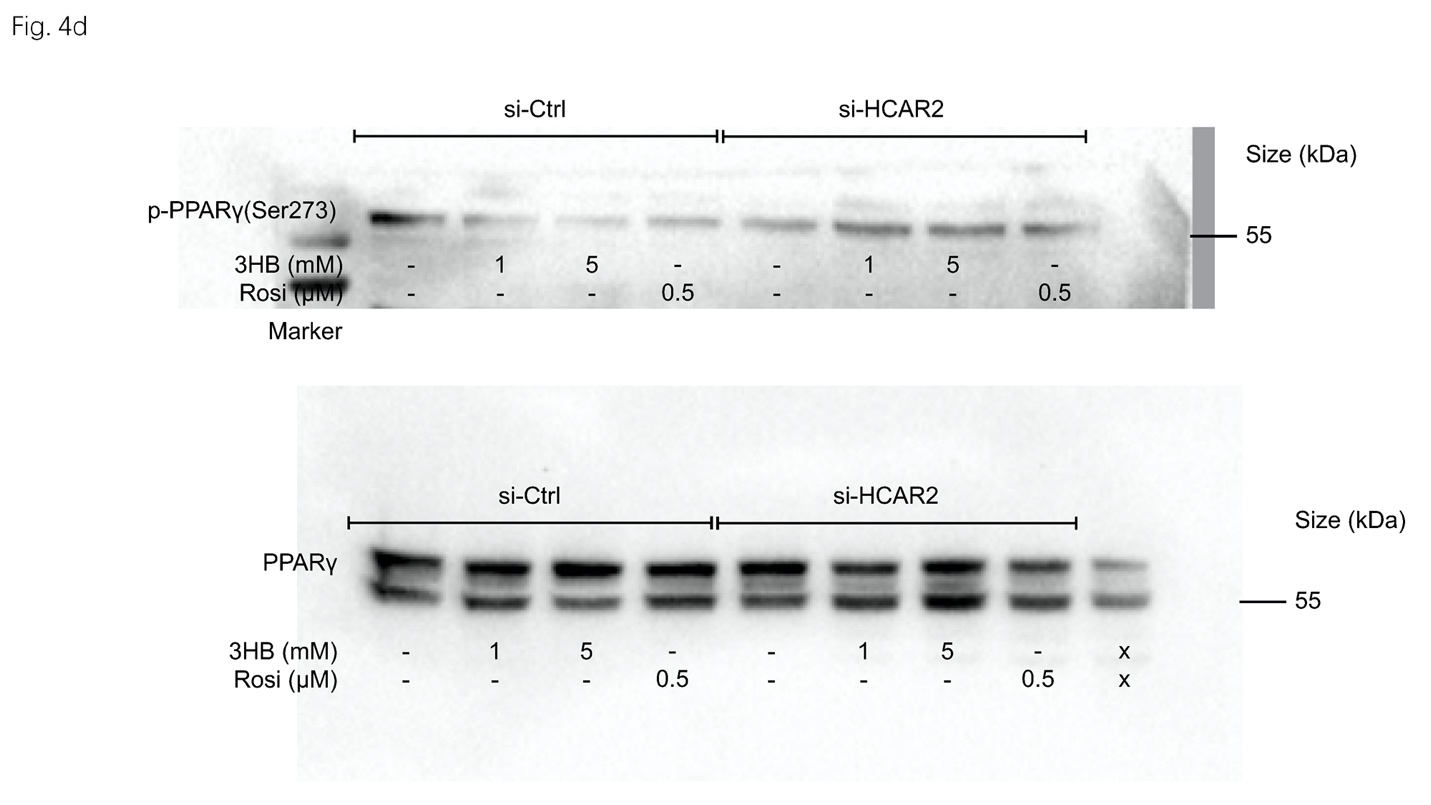


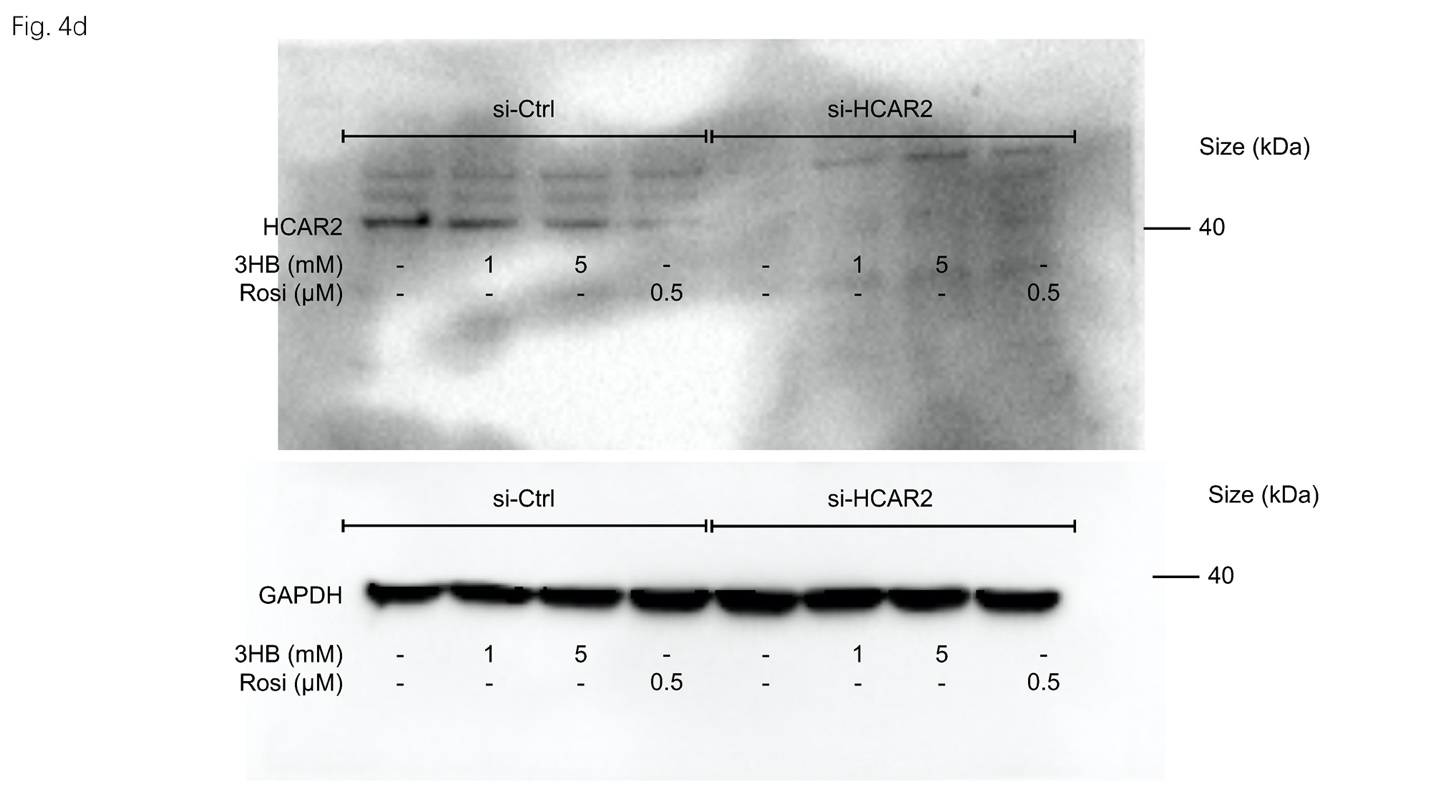


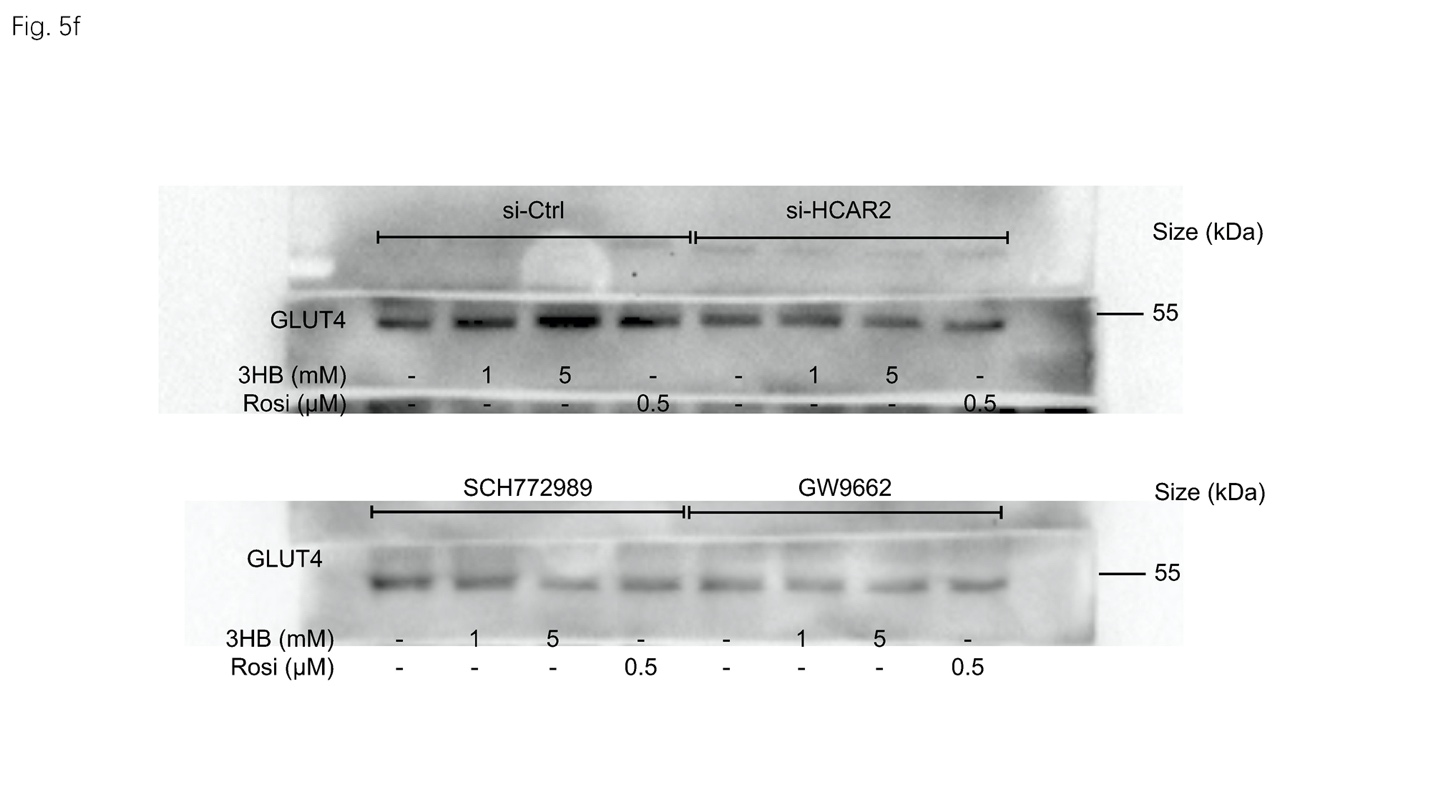

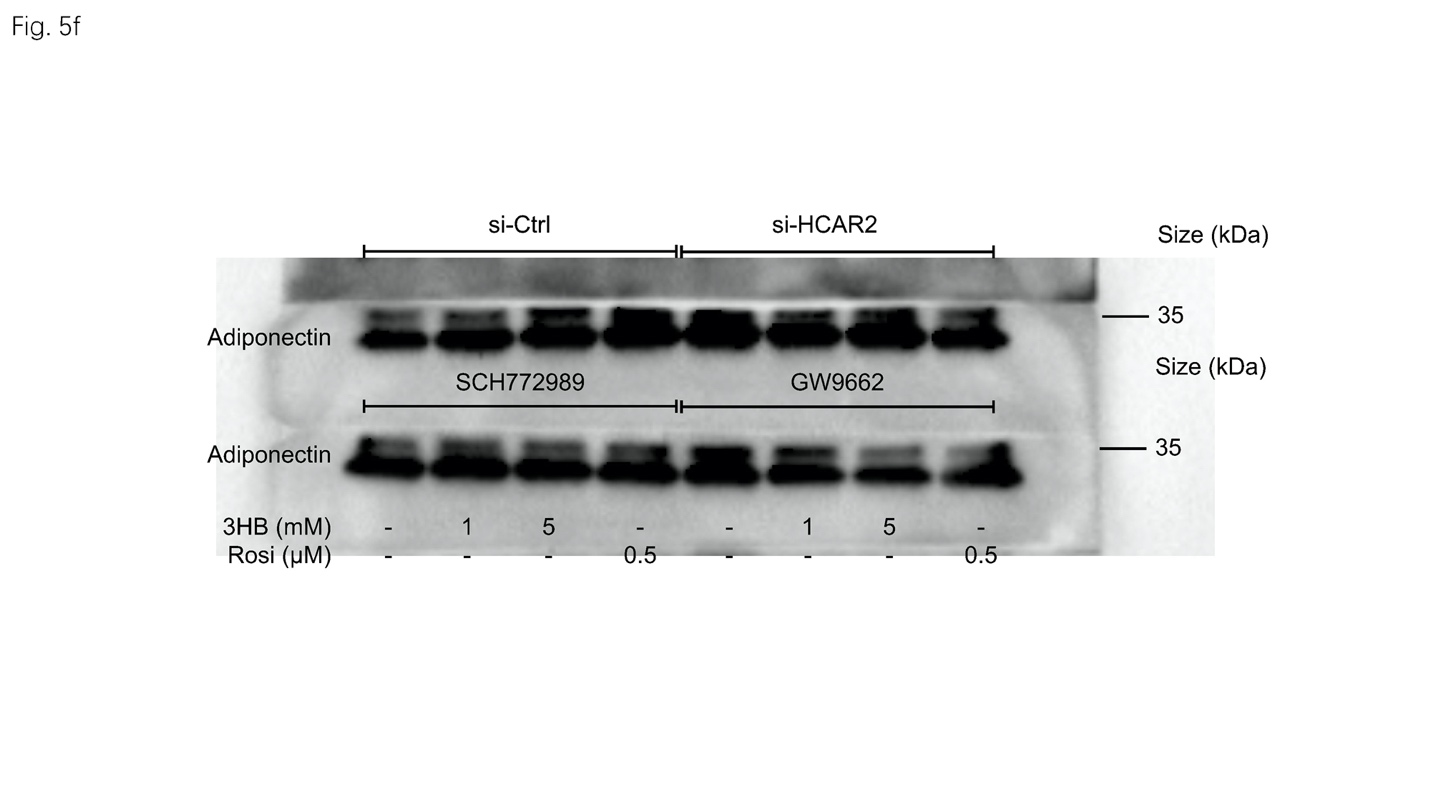


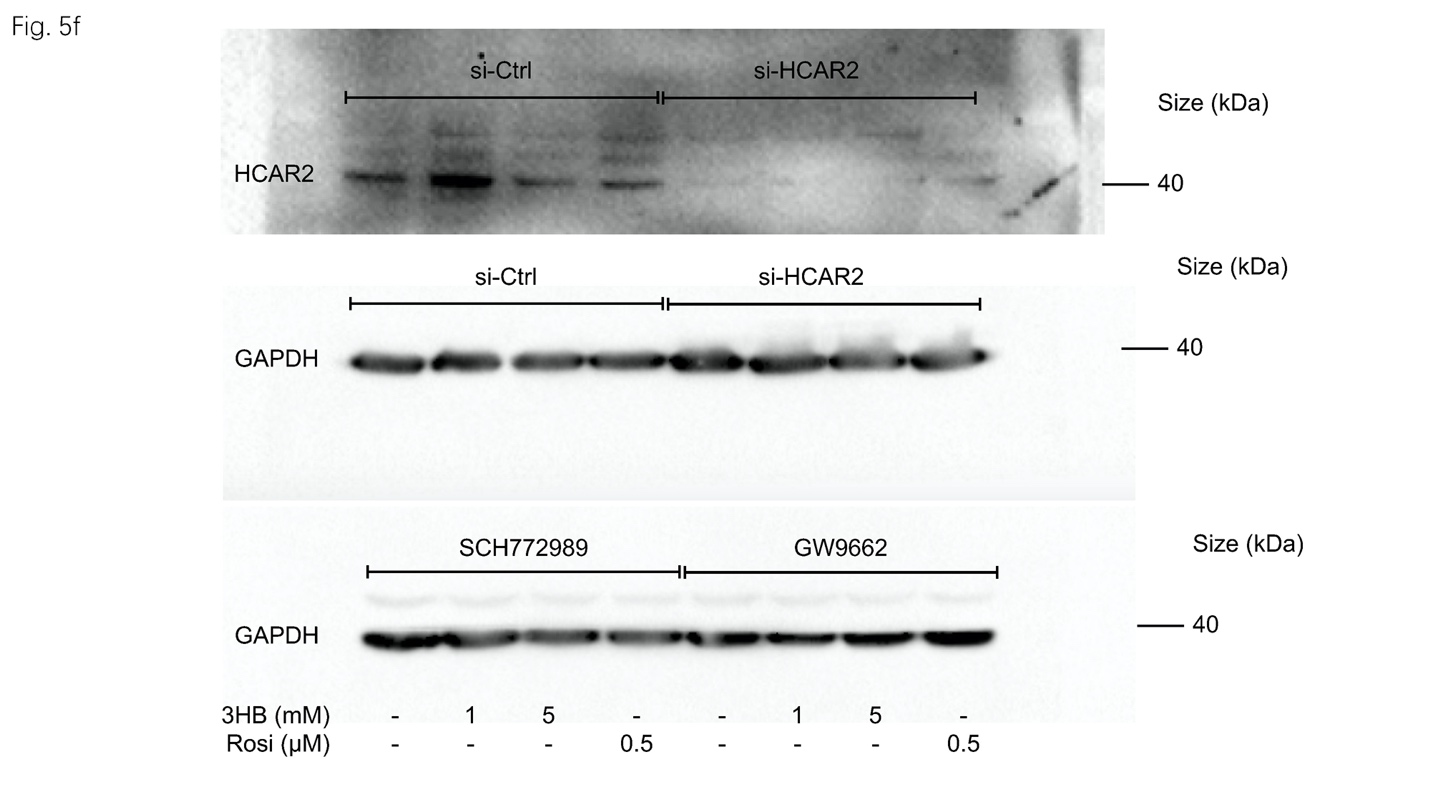


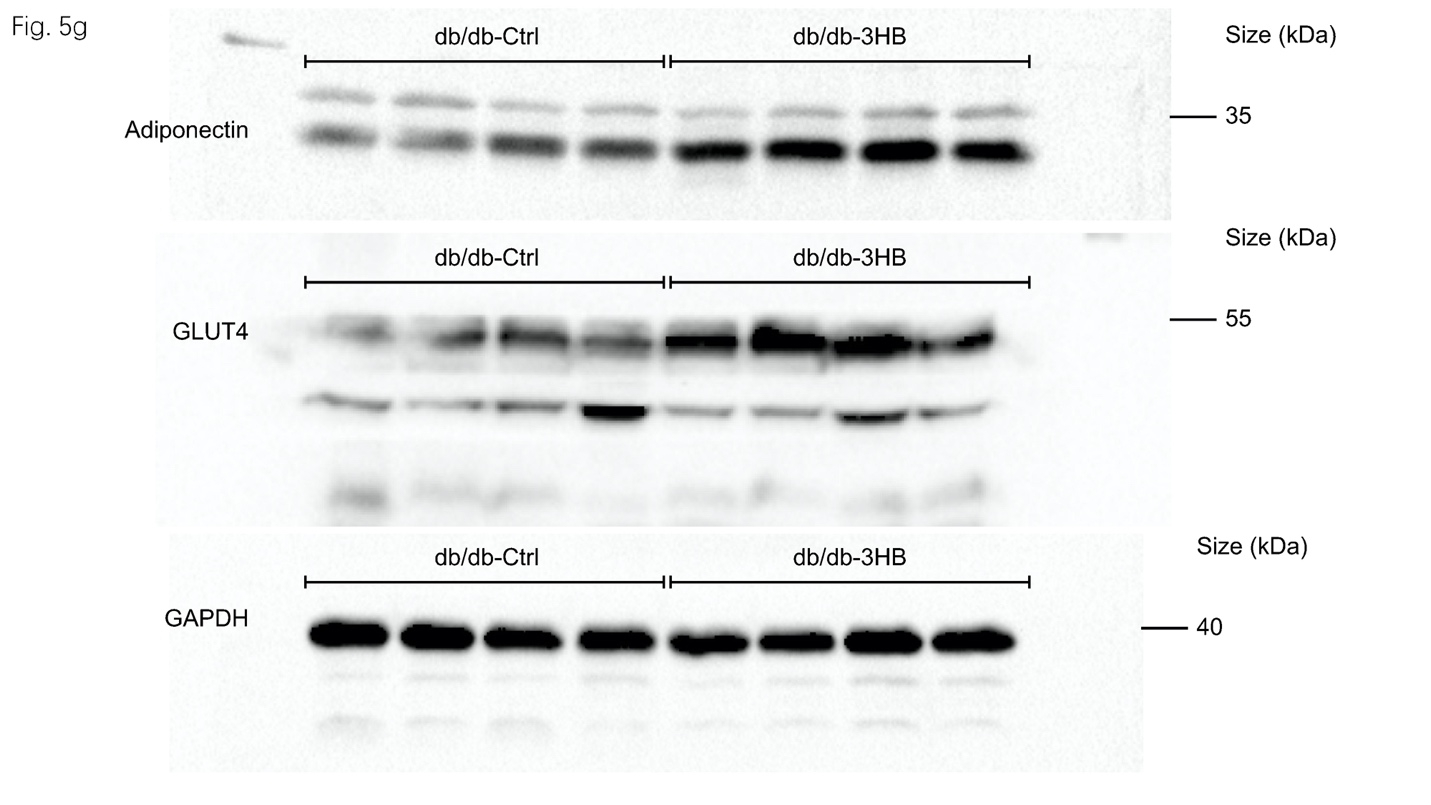

Supplement: Supplementary file 1 — Supplementary Materials for 3-Hydroxybutyrate Ameliorates Insulin Resistance by Inhibiting PPARγ Ser273 Phosphorylation in Type 2 Diabetic Mice [file 41392_2023_1415_MOESM1_ESM.docx]
